# Functionally distinct groups of inherited PTEN mutations in autism and tumour syndromes

**DOI:** 10.1136/jmedgenet-2014-102803

**Published:** 2014-12-19

**Authors:** Laura Spinelli, Fiona M Black, Jonathan N Berg, Britta J Eickholt, Nicholas R Leslie

**Affiliations:** 1 Institute of Biological Chemistry, Biophysics and Bioengineering, School of Engineering and Physical Sciences, Heriot Watt University, Edinburgh, UK; 2 Division of Cell Signalling and Immunology, College of Life Sciences, University of Dundee, Dundee, UK; 3 Clinical Genetics, School of Medicine, University of Dundee, Dundee, UK; 4 Cluster of Excellence NeuroCure and Institute of Biochemistry, Charité—Universitätsmedizin Berlin, Berlin, Germany

**Keywords:** Cancer: breast, Neurosciences, Cell biology

## Abstract

**Background:**

Germline mutations in the phosphatase PTEN are associated with diverse human pathologies, including tumour susceptibility, developmental abnormalities and autism, but any genotype-phenotype relationships are poorly understood.

**Methods:**

We have studied the functional consequences of seven PTEN mutations identified in patients diagnosed with autism and macrocephaly and five mutations from severe tumour bearing sufferers of PTEN hamartoma tumour syndrome (PHTS).

**Results:**

All seven autism-associated PTEN mutants investigated retained the ability to suppress cellular AKT signalling, although five were highly unstable. Observed effects on AKT also correlated with the ability to suppress soma size and the length and density of dendritic spines in primary neurons. Conversely, all five PTEN mutations from severe cases of PHTS appeared to directly and strongly disrupt the ability to inhibit AKT signalling.

**Conclusions:**

Our work implies that alleles causing incomplete loss of PTEN function are more commonly linked to autism than to severe PHTS cases.

## Introduction

Phosphatase and tensin homologue deleted on chromosome ten (PTEN) is a phosphatase that suppresses the activity of the class I phosphoinositide 3-kinase/AKT signalling pathway.^1^ It has been heavily studied due to its status as a tumour suppressor gene in which loss of function mutations are identified in many sporadic tumours and in the germline of patients with diverse phenotypes. Inherited dominant PTEN mutations have been identified in patients with Cowden syndrome and Bannayan-Riley-Ruvalcaba syndrome, conditions that are often grouped together as PTEN hamartoma tumour syndrome (PHTS). They have also been identified in a fraction of patients with autism spectrum disorder (ASD) who also display macrocephaly with and without additional developmental phenotypes characteristic of PHTS.^2–8^ Supporting the causality of PTEN mutations in these phenotypes, a similar tumour spectrum and autism-like phenotypes have been identified in mice either carrying a single null Pten allele or with tissue-specific deletion of Pten.^9–11^

The major diagnostic criteria for PHTS include malignancies of the breast, thyroid and endometrium in addition to benign hamartomas, skin lesions and macrocephaly.^12^ However, the symptoms associated with PTEN mutations are diverse and in some cases, germline mutations have been identified in adult patients only upon presentation with malignancy^13^ ^14^ and in patients with macrocephaly, autism and/or learning disability without further symptoms.^5^ ^15^ A series of clinical and laboratory based studies have presented evidence that mutational functional diversity and genetic background may each contribute to the phenotypical diversity observed in patients carrying PTEN mutations. In some families, individuals carrying the same PTEN mutation have been noted to display very different phenotypes, to the extent of separate diagnoses of Cowden syndrome and Bannayan-Riley-Ruvalcaba syndrome.^16^ ^17^ Additionally, the spectrum of tumours arising in heterozygous mice carrying a null allele of Pten also appears to be strongly dependent on genetic background.^18^ Studies of the mutation types (missense vs truncation) and positions (phosphatase vs C2 domain) within PTEN have argued for^19^ and against^2^ ^20^ genotype-phenotype relationships. On the other hand, evidence that not all PTEN mutations cause one phenotypical profile has been provided by studies of heterozygous mice expressing a stable Pten mutant protein either lacking all phosphatase activity, Pten C124S or that selectively lacks lipid phosphatase activity, Pten G129E. These mice display a more severe tumour phenotype than mice carrying a Pten deletion allele^21^ ^22^ indicating that inactive Pten can aggravate phenotypes, particularly tumour severity, through dominant negative mechanisms. However, whether this represents a consistent genotype-phenotype relationship within PTEN mutation carriers, in particular relating to the occurrence of ASD, is unclear.

## Results

### PTEN missense mutations identified in patients with autism are catalytically competent

**Figure 1:**
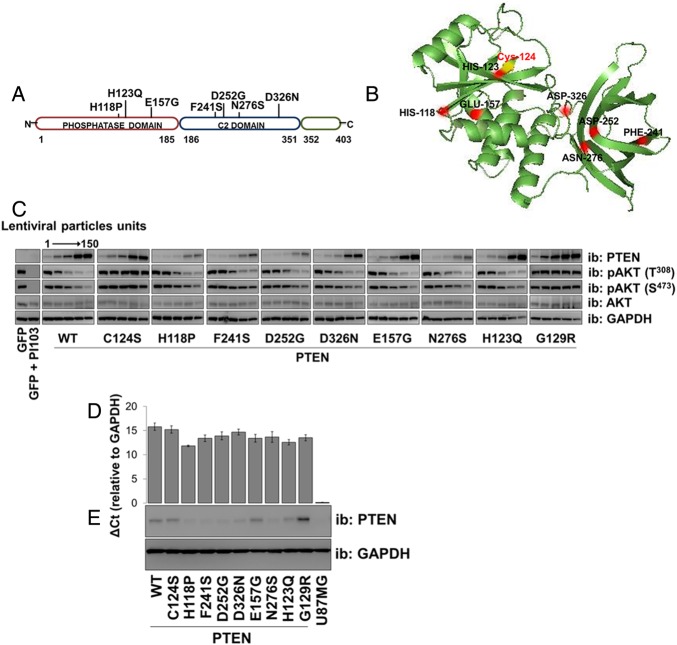
PTEN lipid phosphatase activity. (A) A diagram representing PTEN protein structure and the localisation of the autism-related mutations analysed. (B) PTEN crystal structure indicating mutations. The catalytic Cys-124 is yellow. (C) U87MG cells were transduced with different units (low to high) of lentivirus particles encoding for PTEN WT or PTEN mutants. Control cells were transduced with viruses encoding for GFP and treated with the phosphoinositide 3-kinase (PI3K) inhibitor PI103 (1 µM for 30 min). PTEN expression and AKT phosphorylation were investigated by western blotting of cell lysates. The presented blotting panels for each protein are all from one membrane and are representative of three independent experiments. (D and E) The level of PTEN gene expression was measured by qPCR in U87MG cells transduced with an equal amount of lentivirus particles (two units) encoding for PTEN WT or mutants. (D) Relative PTEN expression was detected using real-time qPCR. Data are shown as mean PTEN ΔCt values relative to GAPDH±SEM. from three experiments each performed in duplicate. (E) Parallel PTEN protein immunoblot. GFP, green fluorescent protein; GAPDH, glyceraldehyde 3-phosphate dehydrogenase.

Recombinant PTEN protein purified from bacteria has been used in many previous studies to demonstrate that phosphatase activity is critical for its tumour suppressor function. In preliminary experiments studying three PTEN mutants identified in patients with autism, we could detect little or no catalytic activity in vitro from protein purified from bacteria, yet transient expression of these mutants in mammalian cells led to a robust suppression of the phosphorylation of the PTEN regulated kinase AKT (see online supplementary figure S1A, B). This apparent difference between assay formats encouraged us to analyse a larger group of seven mutant proteins identified in patients with autism (figure 1A) lacking other manifestations of PHTS.^3–7^ Therefore, we used lentiviruses to express either PTEN wild-type (WT) or individual missense PTEN mutants (C124S, G129R, H118P, H123Q, E157G, F241S, D252G, N276S and D326N) in PTEN-null U87MG glioblastoma cells. These include two recognised active site PTEN mutants that lack catalytic activity: PTEN C124S and PTEN G129R. We expressed PTEN WT and each mutant using five increasing doses of each viral vector (figure 1C). Investigating effects on cellular AKT phosphorylation, when expressed at similar levels, all seven of the autism-related mutants showed similar (or in some cases possibly greater) effects to PTEN WT on AKT phosphorylation, whereas a lack of activity was observed with the inactive mutants PTEN C124S and G129R (figure 1C, D and see online supplementary figure S3A). These data show that the mutations identified in patients with autism do not greatly reduce the ability of the expressed PTEN proteins to inhibit cellular AKT signalling and imply that these mutants retain catalytic activity.

To test their phosphatase activity directly, we overexpressed each protein in U87MG cells and assayed freshly immunopurified PTEN proteins in vitro against radiolabelled phosphatidylinositol 3,4,5-trisphosphate (PIP_3_) vesicles. Recovered activities were weak for many of the PTEN mutants, but this correlated with a low recovery of immunoprecipitated PTEN, probably due to poor stability of these mutants during the immunopurification process (see online supplementary figure S2). In particular, PTEN H118P, F241S, D252G and N276S were recovered poorly and gave very weak activity. Activity normalised to reflect PTEN protein content showed robust activity for all mutants, although weaker than wild type enzyme (see online supplementary figure S2). These experiments also indicate that testing the protein phosphatase activity would be problematic. In addition to this difficulty immunoprecipitating active protein, during these experiments, it was noted that to express most of the autism-related *PTEN* missense mutations at the same level as PTEN WT protein, a higher titre of lentivirus was consistently required (see online supplementary figures S2A and S3A). Therefore, we investigated the possibility that these mutations affected the stability of the PTEN protein.

### Reduced protein stability of autism-associated PTEN mutants

To investigate the relationship between the cellular abundance of PTEN mRNA and protein for the mutants, we expressed PTEN WT and mutants in U87MG cells and measured PTEN mRNA levels by real-time qPCR and protein by western blotting. In all cases, the mRNA abundance corresponded to the titre of lentivirus applied in the experiment. The expression of PTEN WT and the widely used stable mutants, C124S and G129R was robust and similar, as was the expression of two of the autism-associated mutants, H123Q and E157G. However, we observed that for five of the autism-associated mutants (H118P, F241S, D252G, N276S and D326N) the amount of protein expressed was very low (figure 1D, E).

**Figure 2:**
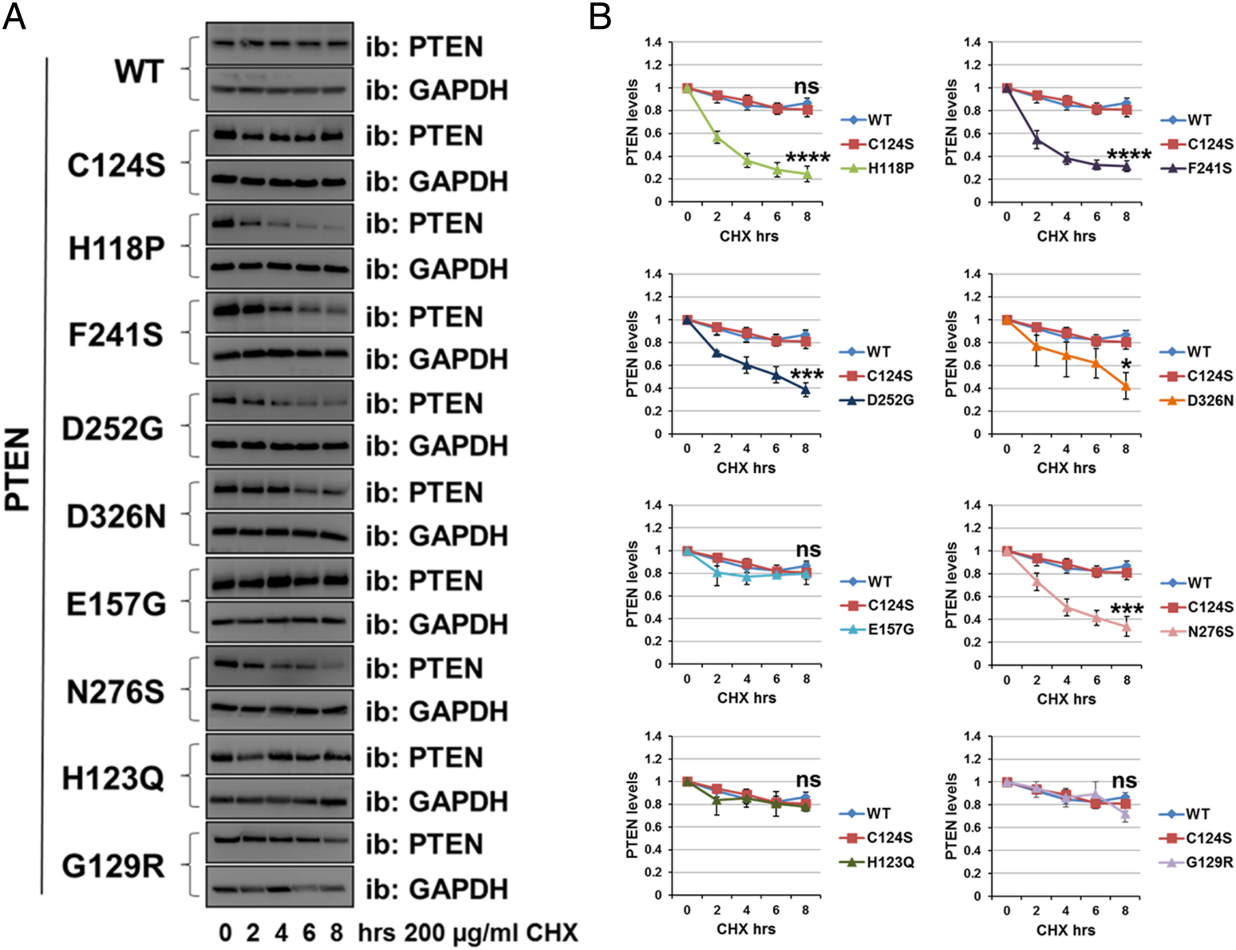
Autism-associated PTEN mutations reduce protein stability. The stability of PTEN mutation proteins was determined using the cycloheximide inhibitor. (A) U87MG cells expressing PTEN WT or PTEN point mutants were treated with 200 µg/mL of the inhibitor cycloheximide for the indicated times, followed by immunoblotting analysis with anti-PTEN and anti-GAPDH antibodies. The blots shown are representative of three experiments. (B) Plot is densitometric quantitation of the cycloheximide chase assay. Data are shown as PTEN level normalised to GAPDH at each time point and compared with that at the 0 time point. The quantitation of the cycloheximide inhibitor assay is derived from three independent experiments (mean±SEM). Six independent experiments for PTEN WT, C124S, H118P and G129R (including the data shown in figure 4D, E). ****p<0.0001, ***p<0.001, **p<0.01, *p<0.05 compared with PTEN WT (Student's t test using GraphPad Prism software).

To assess more directly the stability of these mutant proteins relative to PTEN WT, we used the protein synthesis inhibitor cycloheximide (200 µg/mL). Transduced cells were incubated with the inhibitor for 0 h, 2 h, 4 h, 6 h and 8 h, followed by immunodetection using anti-PTEN antibody (figure 2A, B). Whereas the protein level of PTEN WT and some mutants (C124S, G129R, H123Q and E157G) was reduced by ∼20% after 8 h of inhibition of protein synthesis, the protein level for the PTEN mutants H118P, F241S, D252G, N276S and D326N was decreased by ∼60% after 8 h (figure 2A, B), and was significantly lower than PTEN WT, supporting the hypothesis that these five mutants are unstable.

### Molecular characterisation in primary neurons of the PTEN mutations identified in patients with autism

**Figure 3:**
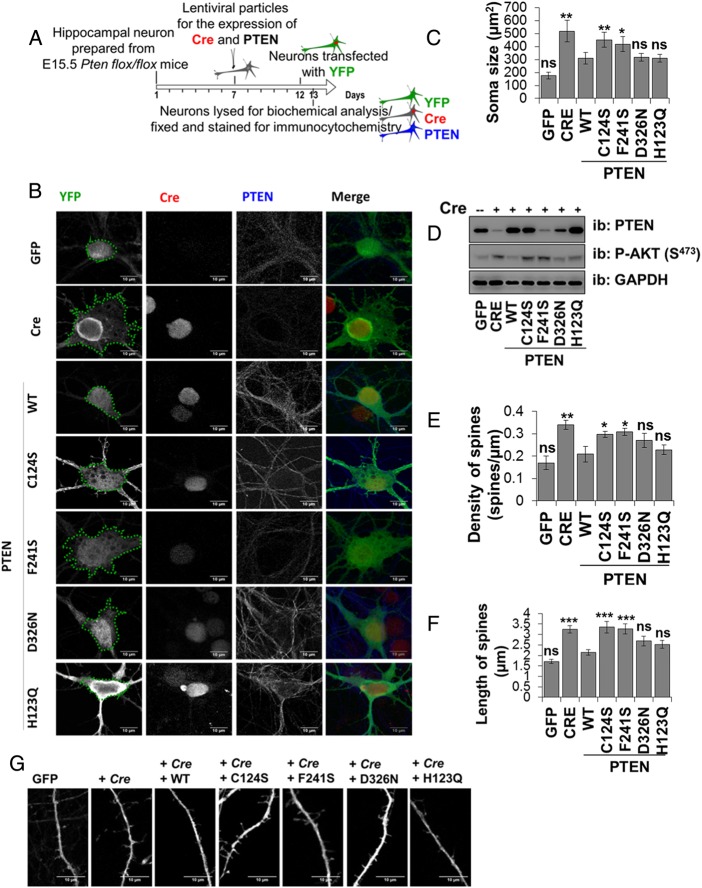
Characterisation of autism-related PTEN mutants in primary neurons. Re-expression at physiological level of PTEN WT and mutants in PTEN knockout neurons is able to support wild type neuronal morphology. (A) Workflow of the experiment. Neurons were transduced after 7 days with viruses encoding Cre recombinase (RFP-Cre) and PTEN WT or mutants as indicated. Cells were lipofected with yellow fluorescent protein (YFP) and then fixed and immunostained 6 days after transduction for GFP (to detect YFP), PTEN and for RFP (RFP-Cre) as showed in the representative images in (B) (scale bars 10 µm). (C) The soma size was measured using ImageJ software and the area measured (green lines) is represented in µm 2±SEM (n=9 cells for each genotype). (D) Parallel neuronal cell lysates were used for western blotting to verify the efficiency of the knockout and the re-expression of PTEN. (E–G) Effects of PTEN expression on spine density and length. (E and F) Quantification of the effects of PTEN genotype on spine density (n=8 dendrites for each genotype) and spines length (n=40 spines measured for each mutant) as mean±SEM. Spines were counted on a dendrite length of 70 µm. ****p<0.0001, ***p<0.001, **p<0.01, *p<0.05 compared with PTEN WT. (Dunnett's post hoc test following a one-way ANOVA using GraphPad Prism software). (G) Representative images of dendritic spines (scale bars 10 µm).

Macrocephaly is one of the several anatomical and cellular abnormalities that have been suggested to be important factors in the pathogenesis of ASD.^23^ Work carried out in mice showed that PTEN can control neuronal morphology and growth, with hypertrophy and dendritic overgrowth and development of macrocephaly and additional behavioural phenotypes similar to human autism observed in mice lacking PTEN in neurons.^10^ ^24–27^ We have therefore used neurons from which endogenous *Pten* is deleted to investigate whether the physiological level re-expression of PTEN WT or autism-associated PTEN mutants is able to support a wild type phenotype of normal dendritic growth and neuronal size. Hippocampal neurons prepared from E15.5 *flox/flox Pten* mice were transduced simultaneously with lentiviruses expressing Cre recombinase (RFP-Cre) to delete endogenous *Pten* and with viruses encoding PTEN WT or PTEN mutants. The mutants PTEN H123Q, F241S and D326N were selected to represent a range of protein stabilities (see figure 2). The deletion of the endogenous *Pten* genes and the re-expression of human PTEN mutants were verified by western blot (figure 3D). Deletion of Pten led to an increase in Akt phosphorylation which was not reduced by the expression of PTEN C124S. The mutant F241S expressed very poorly, however the mutants H123Q and D326N were expressed and able to downregulate Akt phosphorylation as was PTEN WT. These results confirmed that when expressed at physiological levels these mutants are biologically active. We wanted then to verify the effect that they have on soma size and the morphology of dendritic spines by immunocytochemistry. In order to analyse neuronal morphology, 6 days after transduction and 24 h before being fixed and stained, neuronal cultures were lipofected with vectors encoding yellow fluorescent protein to allow the morphological analysis of a transfected neuronal subpopulation (including identifiable transduced and untransduced cells). PTEN WT significantly reduced the soma size (figure 3B, C) of hippocampal neurons, as well as the density and length of dendritic spines (figure 3E–G) to levels similar to control neurons (from which endogenous *Pten* had not been deleted), relative to neurons expressing Cre, with or without additional inactive PTEN C124S. In agreement with the previous results, we found that the autism-related mutants H123Q and D326N were capable of controlling these morphological changes in broad correlation with the amount of protein expressed, showing effect similar to PTEN WT when expressing similar amount of protein. PTEN F241S expressed very poorly and did not affect neuronal signalling or morphology in accordance with its very low apparent stability.

### PTEN mutations identified in severe cases of PHTS lack activity

Considering the view that patients diagnosed only with ASD and macrocephaly may reflect a group at the milder end of the spectrum of phenotypes associated with *PTEN* mutations, we chose to select a comparator group of mutations associated with a severe tumour bearing PHTS phenotype. To do this, we conducted an extensive search of data published in journals and online resources associating specific PTEN mutations with phenotype (see online supplementary tables S1–S3). Although inconsistencies in the categorisation of patients’ severity are likely to be introduced by diversity in the diagnosis and recording of separate phenotypical characteristics between publications, our analysis identified 112 missense mutations that had sufficient associated phenotypical data and which had been identified in a total of 204 patients (see online supplementary tables S2, S3). This analysis shows that missense mutations (rather than truncating mutations) were overrepresented among all PTEN mutation carriers described with ASD compared with all carriers of PTEN mutations, consistent with the hypothesis that more of these mutants may retain some activity (figure S3).

**Figure 4:**
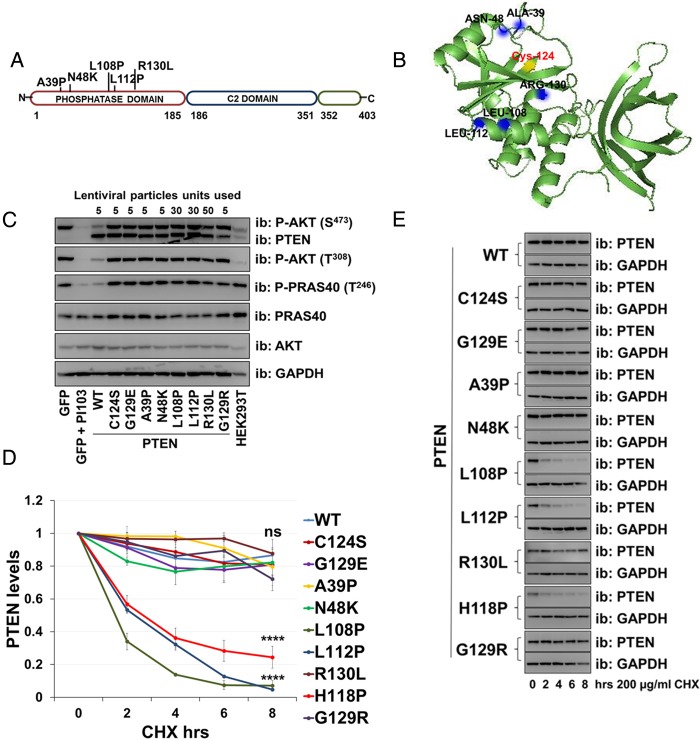
Missense PTEN mutations associated with severe PTEN hamartoma tumour syndrome (PHTS) phenotypes cause complete loss of function. (A) Studied mutations in PTEN associated with PHTS. (B) PTEN crystal structure with catalytic Cys124 in yellow and indicated mutations. (C) PTEN null U87MG cells were transduced with lentiviruses encoding PTEN WT or mutants. Specific lentiviral doses were used for each mutant to standardise PTEN expression level (5 units are equal to 50 µL of viral supernatant). Cell expressing GFP and cells treated with the phosphoinositide 3-kinase (PI3K) inhibitor PI103 (1 µM, 30 min) were controls. PTEN expression and AKT and PRAS40 phosphorylation were investigated by immunoblotting of total cell lysates. (D and E) U87MG cells were transduced with PTEN WT or mutants, treated with 200 µg/mL cycloheximide, lysed at the indicated times and then immunoblotted for PTEN and GAPDH. (D) PTEN protein levels normalised to GAPDH from three independent experiments (mean±SEM). Six independent experiments for PTEN WT, C124S, H118P and G129R (including the data shown in figure 2). ****p<0.0001, ***p<0.001, **p<0.01, *p<0.05 compared with PTEN WT (Student's t test using GraphPad Prism software). (E) The blots shown are representative of three independent experiments.

Five PTEN missense mutations associated with the most severe phenotype (score >6) were selected for characterisation (PTEN A39P, N48K, L108P, L112P and R130L (figure 4A)). These were expressed in U87MG cells together with the already described stable and inactive PTEN mutants C124S, G129R and G129E. The latter two have been identified in Cowden syndrome sufferers and in our classification were associated with a mild/moderate phenotype. When transduced into U87MG cells using lentiviruses, two of these mutant cDNAs (A39P and N48K) were expressed with similar efficiency to WT PTEN but did not suppress cellular AKT phosphorylation (figure 4C). The other three mutants (L108P, L112P and R130L) expressed less efficiency compared with PTEN WT, but when expression was forced to this level, they also failed to downregulate AKT phosphorylation (figure 4C and see online supplementary figure S3B). Analysis of the half-life of PTEN mutant proteins showed that two of the mutations (L108P and L112P) destabilise the protein with a decrease in protein levels by ∼60–80% after 8 h of protein synthesis inhibition, whereas the protein level for the mutants A39P, N48K and R130L, as well as for PTEN WT, C124S, G129E and G129R was reduced by ∼20% (figure 4D, E). This finding that when expressed at the same level as wild type PTEN, all the seven PTEN mutations associated with ASD, but none of the five associated with severe Cowden syndrome analysed was able to regulate AKT is significant (two-tailed Fisher’s exact test p=0.0013).

## Discussion

Our data show that all seven PTEN mutant proteins that we investigated due to their identification in patients with ASD retained the capacity to suppress cellular phosphoinositide 3-kinase-AKT signalling when they were expressed to the same level as wild type PTEN. However, most of these mutant PTEN proteins are unstable and it would be expected that in vivo this instability would lead to a very considerable reduction in protein abundance relative to PTEN WT and a resultant reduction in biological activity. Functional evaluation of PTEN mutants in a yeast based system has previously found significantly lower activity in a set of PHTS/Cowden-associated PTEN mutations than in autism-associated mutations,^28^ although it was unclear whether this effect related to direct inactivating mutations or poor protein stability. It has already been reported for another mutant associated with ASD, PTEN H93R, that this mutation caused a significant reduction in activity in vitro and in cells, to approximately 15% of wild type activity in vitro.^29^ In agreement with our own data, it seems possible that strong but incomplete loss of activity may be related to the observation of ASD symptoms in the absence of some of the more severe phenotypes seen in some patients with Cowden syndrome. In this regard, we have observed mutations that would lead to loss of function through instability and through direct incomplete loss of activity.

In contrast, when a set of PTEN mutations associated with the most severe cases of Cowden syndrome were tested for their cellular activity, all of these five mutant proteins were found to display no detectable effect on AKT phosphorylation even when overexpressed. This implies that complete loss of activity may associate with a more severe PHTS/Cowden phenotype. Of these five proteins, three showed stability similar to wild type PTEN, whereas two were unstable. In the cases of the stable mutants, this supports a hypothesis that the expression of stable inactive protein may exacerbate the phenotype caused by PTEN loss of function, through interference with the functions of the remaining WT PTEN protein and possibly other proteins. Strong evidence supporting the potential importance of this effect comes from the observed phenotypes of Pten knockin mice, in which the most severe tumour phenotype was observed in mice expressing stable PTEN mutants lacking lipid phosphatase activity, G129E or C124S, relative to either mice carrying a Pten deletion or with an unstable PTEN mutation.^21^ ^22^ However, other mechanisms may be involved as this would not seem to explain the occurrence of unstable catalytically inactive PTEN mutants (L108P and L112P) in severe PHTS. Therefore, although the mechanisms mediating these effects are unclear, our work characterises a novel correlation between specific human patient groups and the features of the PTEN mutations they carry.

Our work implies that mutation-specific factors do contribute to the severity of PTEN mutation carriers’ symptoms. These effects may relate to the retention of some biological PTEN activity in the case of patients displaying macrocephaly and ASD in the absence of more severe developmental phenotypes, and to the expression of dominant negative inactive PTEN protein in some cases combining severe developmental and eventually tumour phenotypes. Biochemically, potential dominant negative effects of mutant PTEN proteins have not been extensively studied, although data supporting the functional importance of PTEN dimerisation has very recently been published.^21^ The first trials are underway of potential targeted therapies, such as inhibitors of the mammalian target of rapamycin (mTOR) kinase activated downstream of PTEN loss, to treat patients with inherited PTEN mutations and severe phenotypes. Through a deeper understanding of the signalling mechanisms driving specific phenotypes, it may eventually be possible to tailor treatments to individual patient groups based upon their early symptoms and on an understanding of the cellular effects of defined classes of mutation.

## Materials and methods

### Cell culture

U87MG glioblastoma cells culture, lentivirus preparation and titration were as previously described.^30^ U87MG cells were transduced with lentivirus particles in a medium supplemented with polybrene (16 µg/mL; hexadimethrine bromide, Sigma). Media were changed 24 h after transduction, and after a further 24 h cells were processed. To delete the Pten gene from cultured Pten *flox-flox* murine neurons, a modified lentiviral vector was used in which the expression of the RFP-Cre transgene is driven by a human synapsin-1 promoter.^31^ ^32^ RFP-Cre lentiviruses were produced by triple cotransfection of 6.5 million HEK293 T cells in 75 cm flasks with 10 µg lentiviral vector, 7.5 µg pHR-CMV 8.9 deltaR packaging vector and 5 µg pCMV VSV-G envelop vector using X-tremeGENE 9 DNA transfection reagent (Roche). The 10 mL of viral supernatant was harvested 48 h after transfection, filtered using 0.22 µ filter (Millipore) and the aliquots were snap frozen in liquid nitrogen for 10 min before storing them at −80°C.

### Primary neuron cultures

Hippocampi were isolated from embryonic day 15.5 Pten *flox-flox* mice (E15.5) and digested in trypsin to obtain dissociated cells as described in Kreis *et al*.^33^ Primary hippocampal cells were resuspended in neurobasal medium supplemented with B-27 (serum-free supplement) and cultured on poly-L-ornithine (15 µg/mL, Sigma) coated coverslips at 37°C in 5% CO_2_ for immunocytochemistry and biochemical analysis.

RFP-Cre lentivirus particles were added to the neuronal culture 7 days after seeding, together with lentivirus particles encoding WT or mutant forms of PTEN and left for 6 days. Every 2–3 days half of the medium was replaced by fresh medium. Neuronal cells used for immunocytochemistry were transfected with yellow fluorescent protein 24 h before fixation by using Lipofectamine 2000 as transfection reagent (0.6 µg DNA/0.8 µL Lipofectamine in Optimem). Of the medium 250 µL was removed, collected in a tube, mixed with fresh media in a 1:1 ratio and incubated at 37°C. Transfection mix was added to the cells for 20 min and then removed and replaced by the incubated media. Cells used for biochemical analysis were washed in phosphate buffered saline (PBS) and lysed using RIPA buffer (RIPA lysis and extraction buffer, Thermo Scientific, 25 mM Tris-HCL pH 7.6, 150 mM NaCl, 1% NP-40, 1% sodium deoxycholate, 0.1% sodium dodecylsulphate (SDS), protease inhibitors and phosphatase inhibitors).

## Supplementary Material

Web supplement

## References

[1] Song MS, Salmena L, Pandolfi PP The functions and regulation of the PTEN tumour suppressor. Nat Rev Mol Cell Biol 2012;13:283–96. 22473468 10.1038/nrm3330

[2] Bubien V, Bonnet F, Brouste V, Hoppe S, Barouk-Simonet E, David A, Edery P, Bottani A, Layet V, Caron O, Gilbert-Dussardier B, Delnatte C, Dugast C, Fricker JP, Bonneau D, Sevenet N, Longy M, Caux F. French Cowden Disease N High cumulative risks of cancer in patients with PTEN hamartoma tumour syndrome. J Med Genet 2013;50:255–63. 10.1136/jmedgenet-2012-101339 23335809

[3] Butler MG, Dasouki MJ, Zhou XP, Talebizadeh Z, Brown M, Takahashi TN, Miles JH, Wang CH, Stratton R, Pilarski R, Eng C Subset of individuals with autism spectrum disorders and extreme macrocephaly associated with germline PTEN tumour suppressor gene mutations. J Med Genet 2005;42:318–21. 10.1136/jmg.2004.024646 15805158 PMC1736032

[4] Buxbaum JD, Cai G, Chaste P, Nygren G, Goldsmith J, Reichert J, Anckarsater H, Rastam M, Smith CJ, Silverman JM, Hollander E, Leboyer M, Gillberg C, Verloes A, Betancur C. Mutation screening of the PTEN gene in patients with autism spectrum disorders and macrocephaly. Am J Med Genet B Neuropsychiatr Genet 2007;144B:484–91. 10.1002/ajmg.b.30493 17427195 PMC3381648

[5] McBride KL, Varga EA, Pastore MT, Prior TW, Manickam K, Atkin JF, Herman GE Confirmation study of PTEN mutations among individuals with autism or developmental delays/mental retardation and macrocephaly. Autism Res 2010;3:137–41. 10.1002/aur.132 20533527

[6] Orrico A, Galli L, Buoni S, Orsi A, Vonella G, Sorrentino V Novel PTEN mutations in neurodevelopmental disorders and macrocephaly. Clin Genet 2009;75:195–8. 10.1111/j.1399-0004.2008.01074.x 18759867

[7] Varga EA, Pastore M, Prior T, Herman GE, McBride KL The prevalence of PTEN mutations in a clinical pediatric cohort with autism spectrum disorders, developmental delay, and macrocephaly. Genet Med 2009;11:111–17. 10.1097/GIM.0b013e31818fd762 19265751

[8] Zhou J, Parada LF PTEN signaling in autism spectrum disorders. Curr Opin Neurobiol 2012;22:873–9. 10.1016/j.conb.2012.05.004 22664040

[9] Clipperton-Allen AE, Page DT. Pten haploinsufficient mice show broad brain overgrowth but selective impairments in autism-relevant behavioral tests. Hum Mol Genet 2014;23:3490–505. 10.1093/hmg/ddu057 24497577

[10] Kwon CH, Luikart BW, Powell CM, Zhou J, Matheny SA, Zhang W, Li Y, Baker SJ, Parada LF Pten regulates neuronal arborization and social interaction in mice. Neuron 2006;50:377–88. 10.1016/j.neuron.2006.03.023 16675393 PMC3902853

[11] Stambolic V, Tsao MS, Macpherson D, Suzuki A, Chapman WB, Mak TW High incidence of breast and endometrial neoplasia resembling human Cowden syndrome in pten+/- mice. Cancer Res 2000;60:3605–11. 10910075

[12] Pilarski R, Burt R, Kohlman W, Pho L, Shannon KM, Swisher E Cowden syndrome and the PTEN hamartoma tumor syndrome: systematic review and revised diagnostic criteria. J Nat Canc Inst 2013;105:1607–16. 10.1093/jnci/djt277 24136893

[13] De Vivo I, Gertig DM, Nagase S, Hankinson SE, O'Brien R, Speizer FE, Parsons R, Hunter DJ Novel germline mutations in the PTEN tumour suppressor gene found in women with multiple cancers. J Med Genet 2000;37:336–41. 10.1136/jmg.37.5.336 10807691 PMC1734596

[14] Pradella LM, Evangelisti C, Ligorio C, Ceccarelli C, Neri I, Zuntini R, Amato LB, Ferrari S, Martelli AM, Gasparre G, Turchetti D A novel deleterious PTEN mutation in a patient with early-onset bilateral breast cancer. BMC Cancer 2014;14:70 10.1186/1471-2407-14-70 24498881 PMC3922036

[15] Busa T, Chabrol B, Perret O, Longy M, Philip N Novel PTEN germline mutation in a family with mild phenotype: difficulties in genetic counseling. Gene 2013;512:194–7. 10.1016/j.gene.2012.09.134 23124040

[16] Celebi JT, Tsou HC, Chen FF, Zhang H, Ping XL, Lebwohl MG, Kezis J, Peacocke M Phenotypic findings of Cowden syndrome and Bannayan-Zonana syndrome in a family associated with a single germline mutation in PTEN. J Med Genet 1999;36:360–4. 10353779 PMC1734369

[17] Zori RT, Marsh DJ, Graham GE, Marliss EB, Eng C Germline PTEN mutation in a family with Cowden syndrome and Bannayan-Riley-Ruvalcaba syndrome. Am J Med Genet 1998;80:399–402. 10.1002/(SICI)1096-8628(19981204)80:4<399::AID-AJMG18>3.0.CO;2-O 9856571

[18] Freeman D, Lesche R, Kertesz N, Wang S, Li G, Gao J, Groszer M, Martinez-Diaz H, Rozengurt N, Thomas G, Liu X, Wu H Genetic background controls tumor development in PTEN-deficient mice. Cancer Res 2006;66:6492–6. 10.1158/0008-5472.CAN-05-4143 16818619

[19] Marsh DJ, Coulon V, Lunetta KL, Rocca-Serra P, Dahia PL, Zheng Z, Liaw D, Caron S, Duboue B, Lin AY, Richardson AL, Bonnetblanc JM, Bressieux JM, Cabarrot-Moreau A, Chompret A, Demange L, Eeles RA, Yahanda AM, Fearon ER, Fricker JP, Gorlin RJ, Hodgson SV, Huson S, Lacombe D, LePrat F, Odent S, Toulouse C, Olopade OI, Sobol H, Tishler S, Woods CG, Robinson BG, Weber HC, Parsons R, Peacocke M, Longy M, Eng C Mutation spectrum and genotype-phenotype analyses in Cowden disease and Bannayan-Zonana syndrome, two hamartoma syndromes with germline PTEN mutation. Hum Mol Genet 1998;7:507–15. 10.1093/hmg/7.3.507 9467011

[20] Nelen MR, Kremer H, Konings IB, Schoute F, van Essen AJ, Koch R, Woods CG, Fryns JP, Hamel B, Hoefsloot LH, Peeters EA, Padberg GW Novel PTEN mutations in patients with Cowden disease: absence of clear genotype-phenotype correlations. Eur J Hum Genet 1999;7:267–73. 10.1038/sj.ejhg.5200289 10234502

[21] Papa A, Wan L, Bonora M, Salmena L, Song MS, Hobbs RM, Lunardi A, Webster K, Ng C, Newton RH, Knoblauch N, Guarnerio J, Ito K, Turka LA, Beck AH, Pinton P, Bronson RT, Wei W, Pandolfi PP Cancer-associated PTEN mutants act in a dominant-negative manner to suppress PTEN protein function. Cell 2014;157:595–610. 10.1016/j.cell.2014.03.027 24766807 PMC4098792

[22] Wang H, Karikomi M, Naidu S, Rajmohan R, Caserta E, Chen HZ, Rawahneh M, Moffitt J, Stephens JA, Fernandez SA, Weinstein M, Wang D, Sadee W, La Perle K, Stromberg P, Rosol TJ, Eng C, Ostrowski MC, Leone G Allele-specific tumor spectrum in pten knockin mice. Proc Natl Acad Sci USA 2010;107:5142–7. 10.1073/pnas.0912524107 20194734 PMC2841921

[23] Zhou J, Blundell J, Ogawa S, Kwon CH, Zhang W, Sinton C, Powell CM, Parada LF Pharmacological inhibition of mTORC1 suppresses anatomical, cellular, and behavioral abnormalities in neural-specific Pten knock-out mice. J Neurosci 2009;29:1773–83. 10.1523/JNEUROSCI.5685-08.2009 19211884 PMC3904448

[24] Backman SA, Stambolic V, Suzuki A, Haight J, Elia A, Pretorius J, Tsao MS, Shannon P, Bolon B, Ivy GO, Mak TW Deletion of Pten in mouse brain causes seizures, ataxia and defects in soma size resembling Lhermitte-Duclos disease. Nat Genet 2001;29:396–403. 10.1038/ng782 11726926

[25] Fraser MM, Zhu X, Kwon CH, Uhlmann EJ, Gutmann DH, Baker SJ Pten loss causes hypertrophy and increased proliferation of astrocytes in vivo. Cancer Res 2004;64:7773–9. 10.1158/0008-5472.CAN-04-2487 15520182

[26] Kwon CH, Zhu X, Zhang J, Knoop LL, Tharp R, Smeyne RJ, Eberhart CG, Burger PC, Baker SJ Pten regulates neuronal soma size: a mouse model of Lhermitte-Duclos disease. Nat Genet 2001;29:404–11. 10.1038/ng781 11726927

[27] van Diepen MT, Parsons M, Downes CP, Leslie NR, Hindges R, Eickholt BJ MyosinV controls PTEN function and neuronal cell size. Nat Cell Biol 2009;11:1191–6. 10.1038/ncb1961 19767745 PMC2756284

[28] Rodriguez-Escudero I, Oliver MD, Andres-Pons A, Molina M, Cid VJ, Pulido R A comprehensive functional analysis of PTEN mutations: implications in tumor- and autism-related syndromes. Hum Mol Genet 2011;20:4132–42. 10.1093/hmg/ddr337 21828076

[29] Redfern RE, Daou MC, Li L, Munson M, Gericke A, Ross AH A mutant form of PTEN linked to autism. Protein Sci 2010;19:1948–56. 10.1002/pro.483 20718038 PMC2998728

[30] Davidson L, Maccario H, Perera NM, Yang X, Spinelli L, Tibarewal P, Glancy B, Gray A, Weijer CJ, Downes CP, Leslie NR Suppression of cellular proliferation and invasion by the concerted lipid and protein phosphatase activities of PTEN. Oncogene 2010;29:687–97. 10.1038/onc.2009.384 19915616 PMC2816976

[31] Lois C, Hong EJ, Pease S, Brown EJ, Baltimore D Germline transmission and tissue-specific expression of transgenes delivered by lentiviral vectors. Science 2002;295:868–72. 10.1126/science.1067081 11786607

[32] Xue M, Lin YQ, Pan H, Reim K, Deng H, Bellen HJ, Rosenmund C Tilting the balance between facilitatory and inhibitory functions of mammalian and Drosophila Complexins orchestrates synaptic vesicle exocytosis. Neuron 2009;64:367–80. 10.1016/j.neuron.2009.09.043 19914185 PMC2790209

[33] Kreis P, Hendricusdottir R, Kay L, et al Phosphorylation of the actin binding protein Drebrin at S647 is regulated by neuronal activity and PTEN. *PLoS One* 2013;8:e71957. 10.1371/journal.pone.0071957 PMC3733845 23940795

