# Supplementary material for: Functionally distinct groups of inherited PTEN mutations in autism and tumour syndromes
Source: J Med Genet. 2014 Dec 19;52(2):128–34. doi: 10.1136/jmedgenet-2014-102803 (PMC4316932; doi:10.1136/jmedgenet-2014-102803)
Supplement: Web supplement [file jmedgenet-2014-102803-s1.pdf]

Functionally distinct groups of inherited PTEN mutations in autism and tumour syndromes

Laura Spinelli, Fiona M. Black, Jonathan N. Berg, Britta J. Eickholt and Nicholas R. Leslie

Figure S1

A

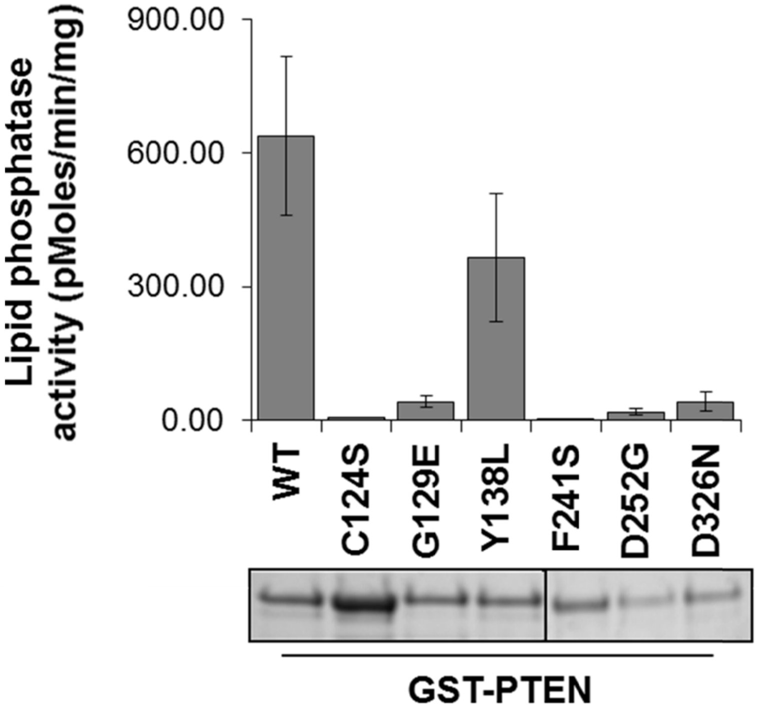

900.00
600.00
300.00
0.00
Lipid phosphatase activity (pMoles/min/mg)
WT
C124S
G129E
Y138L
F241S
D252G
D326N
GST-PTEN

B

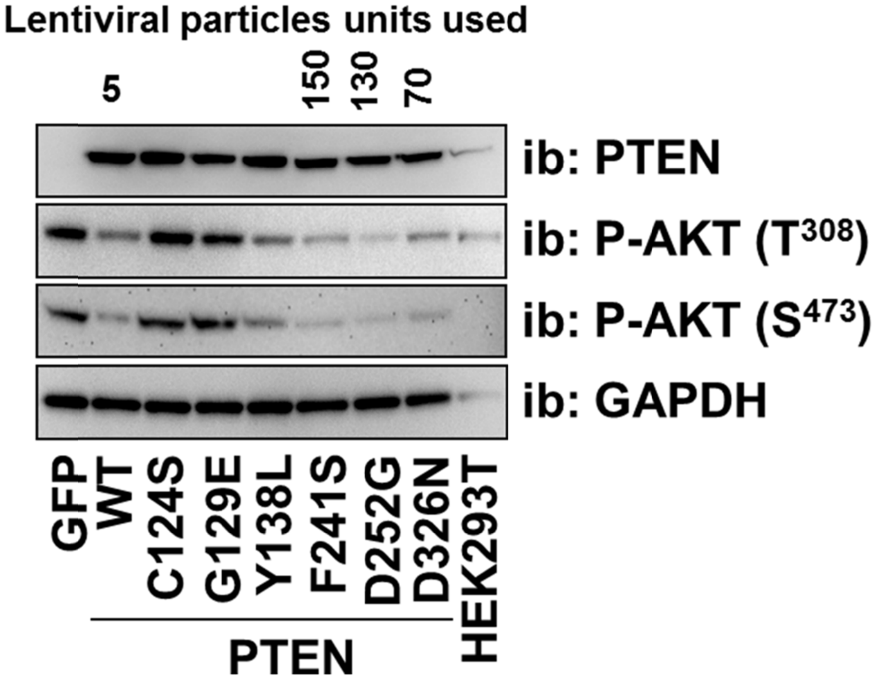

Lentiviral particles units used
5
150
130
70
ib: PTEN
ib: P-AKT (T308)
ib: P-AKT (S473)
ib: GAPDH
GFP
WT
C124S
G129E
Y138L
F241S
D252G
D326N
HEK293T
PTEN

Figure S1. PTEN mutants characterisation in bacteria and U87MG cells

Autism-related mutations proteins drastically reduce PTEN phosphatase activity when expressed in bacteria but they are able to regulate AKT when expressed in U87MG cells. PTEN WT and PTEN mutants (C124S, G129E, Y138L, F241S, D252G and D326N) were expressed in bacteria and purified as GST-fusion proteins. (A) The proteins were separated by SDS-PAGE and stained with Coomassie Brilliant Blue R-250. Proteins were assayed against ^{33}P radiolabelled PIP_3 for 1 hour at 37ºC. The activity is presented as the mean activity ± s.e.m from three experiments performed in duplicates. (B) PTEN null U87MG cells were transduced for 48 hours with lentiviruses encoding GFP, PTEN WT or C124S, G129E, Y138L, F241S, D252G and D326N. PTEN expression and AKT phosphorylation were investigated by western blotting of total cell lysates using total and phospho-specific antibodies. Units of lentiviruses particles used to express PTEN are indicated (5 units are equal to 50 µl of viral supernatant).

Figure S2

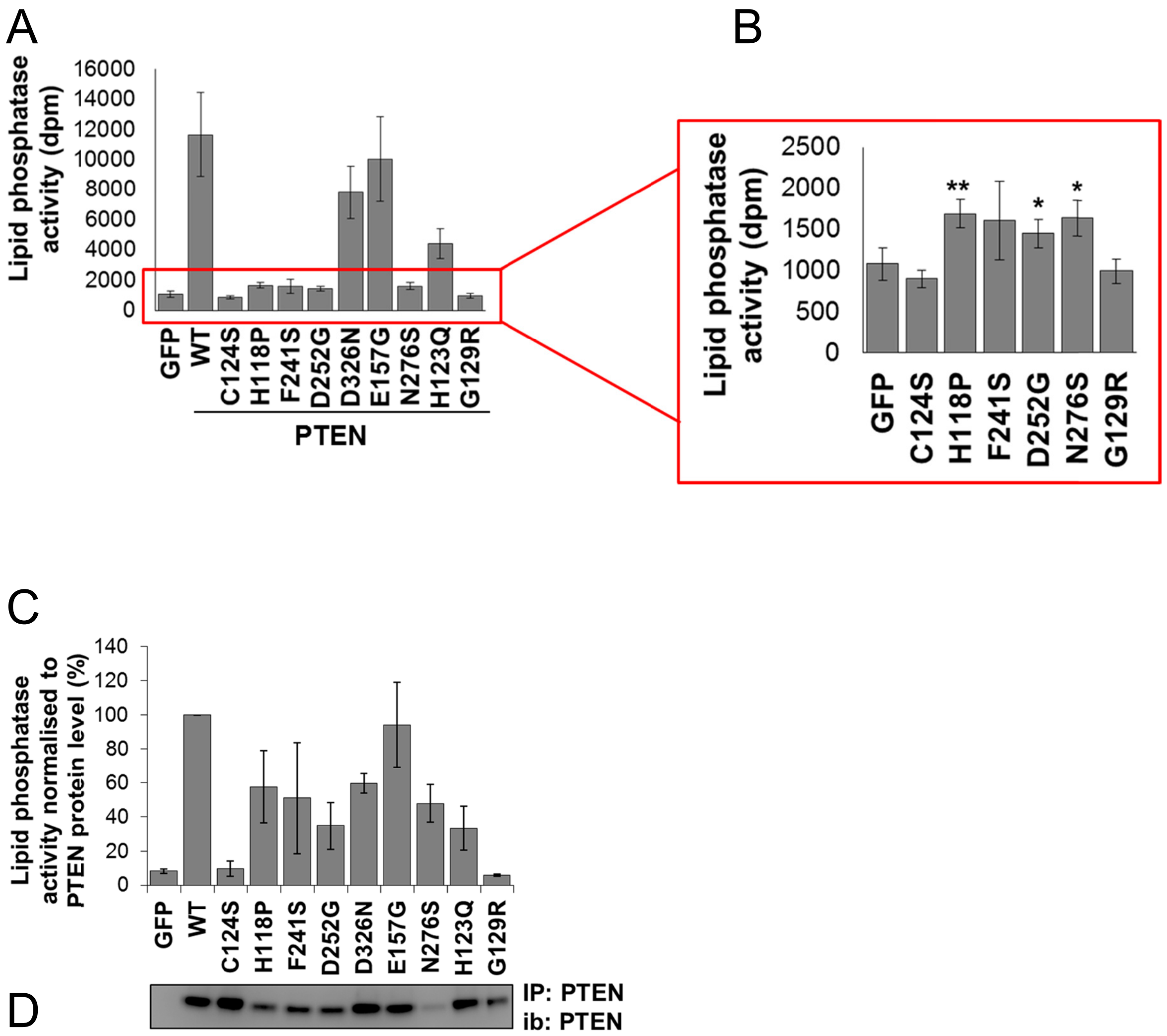
A
B
Lipid phosphatase activity (dpm)
16000
14000
12000
10000
8000
6000
4000
2000
0
GFP
WT
C124S
H118P
F241S
D252G
D326N
E157G
N276S
H123Q
G129R
PTEN
2500
2000
1500
1000
500
**
*
*
C
Lipid phosphatase activity normalised to PTEN protein level (%)
140
120
100
80
60
40
20
D
IP: PTEN
ib: PTEN

Figure S2. Raw data from the PTEN lipid phosphatase activity assay

PTEN proteins or GFP were transiently over-expressed in U87MG cells and after cell lysis, immunoprecipitated using an antibody raised against the N-terminus of PTEN. Immune complexes were assayed against radiolabelled PIP_3 vesicles. (A) Mean raw phosphatase activity in dpm released phosphate is shown, derived from six experiments performed on different days, ± s.e.m.

(B) This shows the same mean raw phosphatase activity data as (A) rescaled to show the differences between raw values with low recovered activity. Across the six experiments there was no overlap between the released phosphate values for any of the seven mutants and the data obtained using the inactive PTEN C124S control. Accordingly, for six of the seven mutants the measured released phosphate was significantly higher than detected with this inactive control (the exception being F241S). (**$p< 0.01$, *$p<0.05$ compared to PTEN C124S; Student's *t*-test using Excel and GraphPad Prism software). (C) This shows the mean activity from six independent experiments ± s.e.m as a % of wild-type PTEN activity after normalisation to the amount of protein immunoprecipitated. Immunoprecipitated protein was determined by densitometry (ImageQuant TL software) after immunoblotting and direct chemiluminiscence detection using CCD camera. (D) A representative immunoblot of the immunoprecipitated proteins from one of the experiments.

Figure S3

A

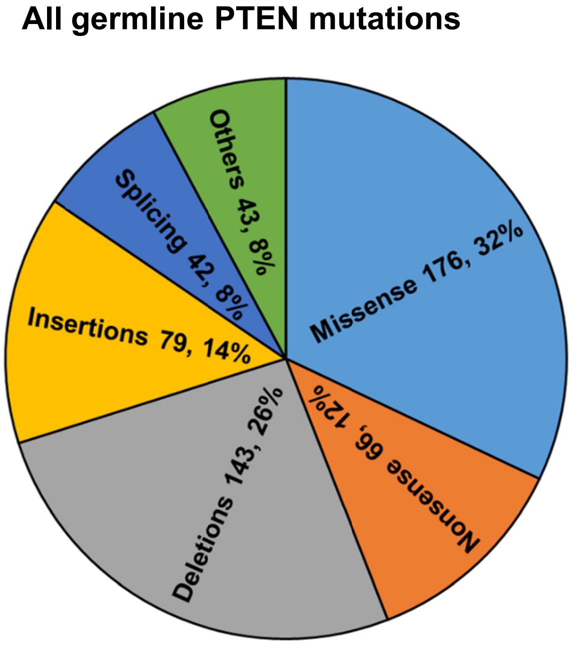

B

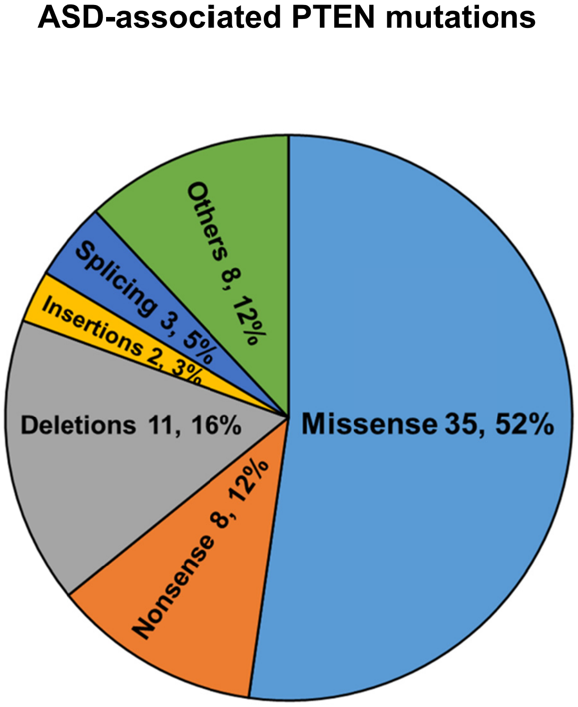

Figure S3. Classification of all inherited PTEN mutations (A) and selectively those associated with ASD (B). The germline PTEN mutations detailed in Tables S1, S3 and S4 were classified into the categories shown. (A) displays the number of apparently independent patients carrying PTEN mutations and (B) the classification of PTEN mutations in independent patients described with ASD, either in the presence or absence of other symptoms including PHTS. A significantly larger fraction of ASD associated mutations are missense mutations 35/67 and 176/549 ($p<0.005$, Fishers Exact Test)

Figure S4

A

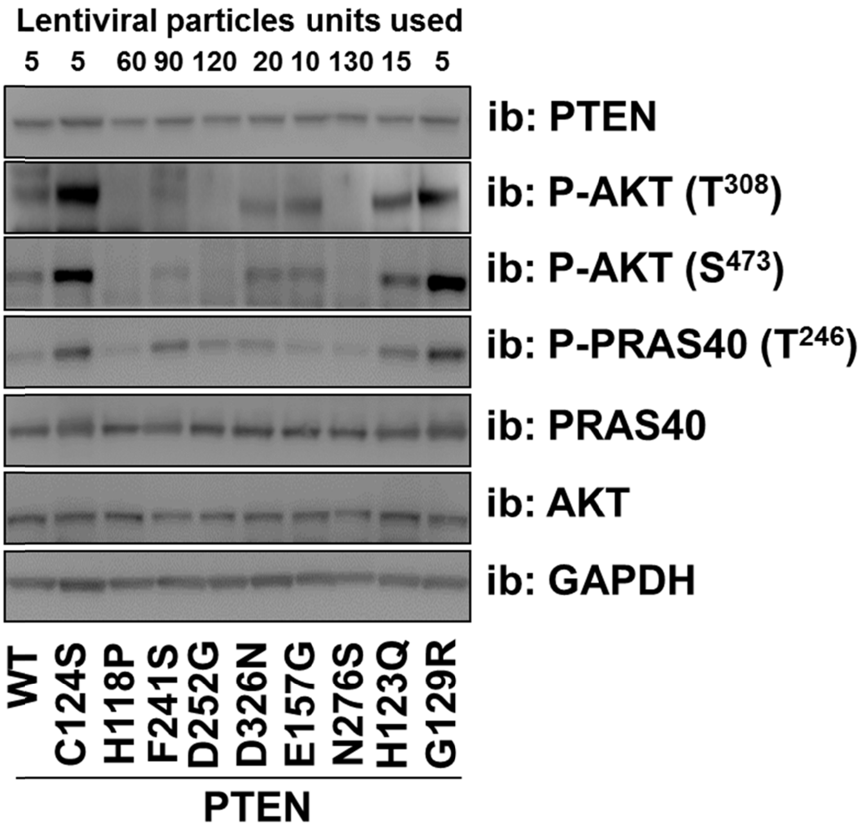
Lentiviral particles units used
5 5 60 90 120 20 10 130 15 5
ib: PTEN
ib: P-AKT (T308)
ib: P-AKT (S473)
ib: P-PRAS40 (T246)
ib: PRAS40
ib: AKT
ib: GAPDH
WT
C124S
H118P
F241S
D252G
D326N
E157G
N276S
H123Q
G129R
PTEN

B

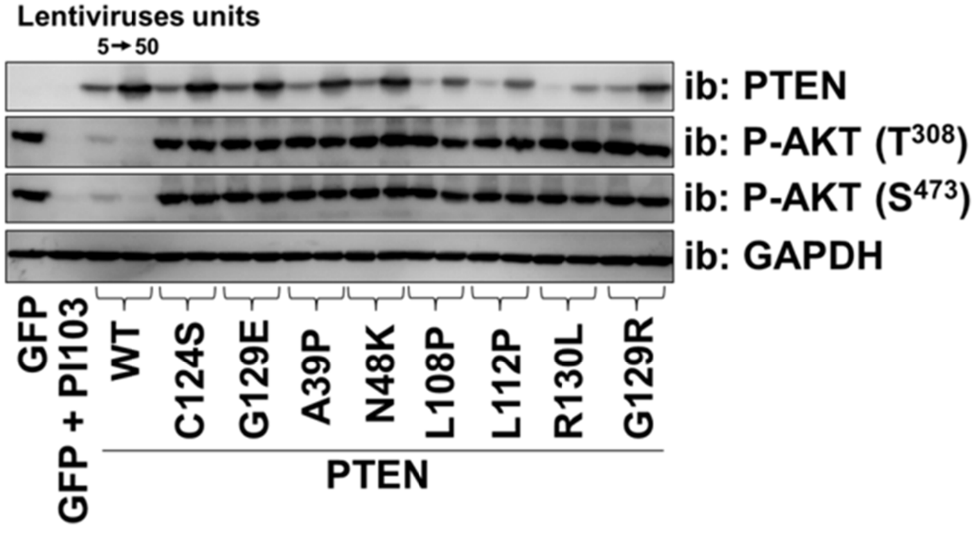
Lentiviruses units
5→50
ib: PTEN
ib: P-AKT (T308)
ib: P-AKT (S473)
ib: GAPDH
GFP
GFP + PI103
WT
C124S
G129E
A39P
N48K
L108P
L112P
R130L
G129R
PTEN

Figure S4. Regulation of AKT and downstream pathways by autism- and Cowden-related mutant proteins in U87MG cells

PTEN null U87MG cells were transduced for 48 hours with lentiviruses encoding PTEN WT or mutants using (A) different amount of lentiviral particles, as shown, for each mutant in order to obtain the same PTEN expression level or (B) two concentrations (low and high) of lentiviruses particles (5 units are equal to 50 µl of viral supernatant). Cell expressing GFP and cells treated with the PI3K inhibitor PI103 (1 µM for 30 minutes) were used as control. The evaluation of PTEN and phosphorylated and total AKT and PRAS40 in the lysates from transduced cells was carried out by western blotting using total and phospho-specific antibodies. The blotting panels shown are representative of three independent experiments.

Table S1. PTEN mutations associated with ASD

Missense	Nonsense	Deletions	Insertions	Splicing	Others	References
**c.278A>G H93R**						Butler et al., 2005
**c.722T>C F241S**						Butler et al., 2005
**c.755A>G D252G**						Butler et al., 2005
**c.66C>G D22E**						Buxbaum et al., 2007
					c._1088C>T	Buxbaum et al., 2007
					c._1084C>T	Buxbaum et al., 2007
					c. IVS3-9T>C	Buxbaum et al., 2007
**c.976G>A D326N**						Buxbaum et al., 2007
					c._1026C>A	Buxbaum et al., 2007
					c._903G>A	Buxbaum et al., 2007
**c.353A>C H118P**						Orrico et al., 2009
**c.530T>G Y176C**						Orrico et al., 2009
**c.824A>G N276S**						Orrico et al., 2009
			c.519-520insT			Varga et al., 2009
	c.388C>T R130*					Varga et al., 2009
	c.416T>A L139*					Varga et al., 2009
**c.470A>G E157G**						Varga et al., 2009
**c.369C>G H123Q**						McBride et al., 2010
**c.518G>A R173H**						McBride et al., 2010
**c.232A>G T78A**						Schaaf et al., 2011
**c.75G>T L25F**						Mester et al., 2012
	c.1003C>T R335*					Mester et al., 2012
		Whole gene deletion				Mester et al., 2012
			c.420-421insA			Mester et al., 2012
**c.392C>T T131I**						O'Roak et al., 2012
**c.500C>A T167N**						O'Roak et al., 2012
		c.158-159delTA				Bubien et al., 2013
		c.284delC				Bubien et al., 2013

Missense	Nonsense	Deletions	Insertions	Splicing	Others	References
	c.388C>T R130*					Bubien et al., 2013
	c.388C>T R130*					Bubien et al., 2013
**c.392C>T T131I**						Bubien et al., 2013
**c.403A>G I135V**						Bubien et al., 2013
**c.406T>C C136R**						Bubien et al., 2013
**c.518G>A R173H**						Bubien et al., 2013
		c.586delC				Bubien et al., 2013
		c.586delC				Bubien et al., 2013
		c.586delC				Bubien et al., 2013
				c.635-1G>C		Bubien et al., 2013
				c.635-3C>G		Bubien et al., 2013
**c.830C>G T277R**						Bubien et al., 2013
		c.1004-1005delGA				Bubien et al., 2013
**c.202T>A Y68N**						Klein et al., 2013
**c.113C>A P38H**						Klein et al., 2013
**c.764T>C V255A**						Klein et al., 2013
	c.959T>G L320*					Vanderver et al., 2014
**c.17A>T L6I**						Vanderver et al., 2014
**c.194A>G Y65C**						Vanderver et al., 2014
**c.511C>G Q171E**						Vanderver et al., 2014
	c.388C>T R130*					Vanderver et al., 2014
**c.737C>T P246L**						Vanderver et al., 2014
**c.633C>G C211W**						Vanderver et al., 2014
		c.43delA				Marchese et al., 2014
**c.208C>G L70V**						Hobert et al., 2014
					c.3G>T M1I	Hobert et al., 2014
					c.3G>T M1I	Hobert et al., 2014
	c.1003C>T R335*					Hobert et al., 2014
				c.209+5G>A		Hobert et al., 2014

Missense	Nonsense	Deletions	Insertions	Splicing	Others	References
**c.40C>G R14G**						Frazier et al., 2014
**c.755A>T D252V**						Frazier et al., 2014
**c.202T>C Y68H**						Frazier et al., 2014
**c.37A>C N12T**						Frazier et al., 2014
**c.395G>A G132D**						Frazier et al., 2014
**c.277C>T H93Y**						Frazier et al., 2014
		c.597delGTT				Frazier et al., 2014
					c._1177C>T	Frazier et al., 2014
		4-Mb deletion				Frazier et al., 2014
		10q deletion				Frazier et al., 2014

Tables S2 and S3. Severity index scoring system.

Based on the clinical description of the patients carrying PTEN germline mutations, the severity scores were created according to the number of sites affected as well as the presence of mental retardation/ development delay (MR/DD). The presence of macrocephaly was recorded but not included in the severity index score, as macrocephaly was seen in almost every subject, with the chance that it was not recorded in others. For each site affected by benign tumours or lesions it was assigned one point, two points for each site affected by malignant tumours, and one point for the presence of mental retardation. The severity index score is the sum of these numbers. Patients who scored one to three were classified as “mild”, four to five as “moderate” and six or above as “severe”.

The choice of mutations to analyse has been made based on the “index score average” resulting from the average of the scores of all patients carrying the same PTEN mutation. It should be noted that the characterised PTEN mutants associated with severe phenotypes (see figure 4) were selected at a mid-point of the progression of the project and that the data tabulated here contain very recently published information added since that time. Efforts have been made to minimise the chances of double counting but this cannot be excluded.

Table S2

Severity index scoring system			
**Site affected**	**Benign lesion/tumour**	**Malignant tumour/cancer**	**MR/DD**
**CNS**	**1 point** assigned for each site affected	**2 points** assigned for each site affected	**1 point** assigned for the presence of Mental Retardation/ Developmental Delay
**Thyroid**			
**Breast**			
**Skin/Mucosa**			
**Gastrointestinal tract**			
**Others**			
**Total score**	**1-3 MILD**<br>**4-5 MODERATE**<br>**6 + SEVERE**		

Table S3

Mutations	Index score average	Index score patients	Assigned Phenotype	References
**M1I**	1	1	ASD	[1] Hobert et al., 2014
		1	ASD	[1] Hobert et al., 2014
**K6I**	1	1	ASD	[2] Vanderver et al., 2014
**K6E**	1	1	ASD	[2] Vanderver et al., 2014
**K13E**	3	4	CS	[3] Bubien et al., 2013
		4	CS	[3] Bubien et al., 2013
		1	CS	[3] Bubien et al., 2013
**R14G**	0	0	ASD	[4] Frazier et al., 2014
**R15S**	3	3	CS	[5] Nagy et al., 2011
**Y16H**	4	4	CS	[6] Ngeow et al., 2014
**D22E**	0	0	ASD	[7] Buxbaum et al., 2007
**D24Y**	5	2	BRRS	[8] Celebi et al., 1999
		8	CS	[3] Bubien et al., 2013
**D24G**	4	4	CS	[6] Ngeow et al., 2014
**D24H**	6	6	CS	[6] Ngeow et al., 2014
**D24V**	5	5	CS	[6] Ngeow et al., 2014
**L25F**	0	0	ASD	[9] Mester et al., 2012
**T26P**	2	2	Unknown	[9] Mester et al., 2012
**Y27N**	4	4	CS	[6] Ngeow et al., 2014
**Y27C**	1	1	Unknown	[2] Vanderver et al., 2014
**P30L**	1	1	CS	[3] Bubien et al., 2013
**A34D**	2	2	BRRS	[10] Marsh et al., 1999
		2	BRRS	[10] Marsh et al., 1999
**M35R**	1	1	JPC	[11] Olschwang et al., 1998
**M35T**	4	4	PLS	[12] Zhou et al., 2001
**G36R**	5	5	CS	[13] Celebi et al., 2000
**P38H**	1	1	ASD	[14] Klein et al., 2013
**A39P**	7	7	CS	[15] Tate et al., 2008
**G44D**	2	2	Unknown	[16] Varga et al., 2009
**N48K**	8	8	CS	[17] Vega et al., 2003
**I50T**	1	1	Unknown	[2] Vanderver et al., 2014
**H61D**	0	0	VATER	[18] Reardon et al., 2001
**H61R**	4	4	CS	[6] Ngeow et al., 2014
**Y65C**	0	0	ASD	[2] Vanderver et al., 2014
		0	ASD	[2] Vanderver et al., 2014
**I67R**	7	7	CS	[3] Bubien et al., 2013
**Y68N**	2	2	ASD	[14] Klein et al., 2013
**Y68D**	0.5	1	PS	[19] Loffeld et al., 2006
		0	CS	[19] Loffeld et al., 2006
**Y68H**	1.33	1	BRRS	[20] Marsh et al., 1998
		3	CS	[20] Marsh et al., 1998
		0	ASD	[4] Frazier et al., 2014
**Y68C**	2	2	CS	[21] Lobo et al., 2009
**L70P**	2	2	CS	[20] Marsh et al., 1998
**L70V**	3	3	ASD	[1] Hobert et al., 2014
**T78A**	0	0	ASD	[22] Schaaf et al., 2011
**A79T**	3	3	BC	[23] Figer et al., 2002
**N82T**	3	3	BC	[23] Figer et al., 2002
**Y88C**	1	1	PHTS	[9] Mester et al., 2012
**D92A**	5	5	CS	[3] Bubien et al., 2013
**H93Y**	2.66	4	CS	[24] Kohno et al., 1998
		4	CS	[6] Ngeow et al., 2014
		0	ASD	[4] Frazier et al., 2014

Mutations	Index score average	Index score patients	Assigned Phenotype	References
**H93R**	1	1	ASD	[25] Butler et al., 2005
**P96Q**	2.5	3	CS	[26] Bussaglia et al., 2002
		2	CS	[26] Bussaglia et al., 2002
**P96R**	3.5	3	CS	[9] Mester et al., 2012
		4	CS	[27] Busch et al., 2013
**C105Y**	2	2	BRRS	[10] Marsh et al., 1999
**D107G**	1	1	Unknown	[2] Vanderver et al., 2014
**L108P**	6.7	6	PHTS	[28] Tan et al., 2007
		7	PHTS	[3] Bubien et al., 2013
		7	CS	[29] Ngeow et al., 2013
**L112P**	11	11	CS	[30] Tsou et al., 1998
**H118P**	1	1	ASD	[31] Orrico et al., 2009
**V119I**	4	4	Multiple cancers	[32] De Vivo et al., 2000
		4	Multiple cancers	[32] De Vivo et al., 2000
**H123Q**	2.5	1	ASD	[33] McBride et al., 2010
		4	CS	[34] Kersseboom et al., 2012
**H123R**	4	4	CS	[35] Nelen et al., 1997
**H123D**	3	3	CS	[26] Bussaglia et al., 2002
**C124R**	4	2	CS	[35] Nelen et al., 1997
		4	CS	[35] Nelen et al., 1997
		6	CS	[35] Nelen et al., 1997
**A126P**	3	3	CS	[9] Mester et al., 2012
**K128E**	4	4	CS	[27] Busch et al., 2013
**K128N**	3	3	CS	[29] Ngeow et al., 2013
**G129R**	3.5	5	CS	[36] Elia et al., 2012
		2	Unknown	unpublished patient
**G129E**	4	4	CS	[37] Liaw et al., 1997
		5	CS	[37] Liaw et al., 1997
		7	CS	[37] Liaw et al., 1997
		2	CS	[37] Liaw et al., 1997
		2	CS	[29] Ngeow et al., 2013
**R130Q**	3.9	5	CS	[38] Kurose et al., 1999
		4	CS/BRRS	[38] Kurose et al., 1999
		3	CS/BRRS	[38] Kurose et al., 1999
		3	CS/BRRS	[38] Kurose et al., 1999
		5	CS	[39] Heindl et al., 2012
		4	CS	[39] Heindl et al., 2012
		5	CS	[29] Ngeow et al., 2013
		4	CS	[6] Ngeow et al., 2014
		2	CS	[6] Ngeow et al., 2014
**R130G**	1.8	2	CS	[21] Lobo et al., 2009
		4	Unknown	[9] Mester et al., 2012
		1	CS	[3] Bubien et al., 2013
		1	CS	[3] Bubien et al., 2013
		1	Unknown	[3] Bubien et al., 2013
		2	CS	[3] Bubien et al., 2013
**R130L**	5	3	CS	[3] Bubien et al., 2013
		3	CS	[3] Bubien et al., 2013
		10	CS	[20] Marsh et al., 1998
		4	CS	[6] Ngeow et al., 2014
**T131I**	1.7	1	CS/ASD	[3] Bubien et al., 2013
		3	Unknown	[3] Bubien et al., 2013
		1	ASD	[40] O'Roak et al., 2012
**G132A**	1	1	PHTS	[28] Tan et al., 2007

Mutations	Index score average	Index score patients	Assigned Phenotype	References
**G132D**	3.16	2	CS	[41] Derrey et al., 2004
		6	CS	[3] Bubien et al., 2013
		4	CS	[3] Bubien et al., 2013
		3	CS	[3] Bubien et al., 2013
		4	CS	[39] Heindl et al., 2012
		0	ASD	[4] Frazier et al., 2014
**G132V**	3	3	PHTS	[42] Tekin et al., 2006
**M134R**	0.75	0	BRRS	[23] Figer et al., 2002
		0	BRRS	[23] Figer et al., 2002
		0	BRRS	[23] Figer et al., 2002
		3	Unknown	[9] Mester et al., 2012
**M134I**	0	0	Unknown	[43] Busa et al., 2013
		0	Unknown	[43] Busa et al., 2013
		0	Unknown	[43] Busa et al., 2013
		0	Unknown	[43] Busa et al., 2013
**M134T**	4.66	1	MR	[33] McBride et al., 2010
		5	CS	[29] Ngeow et al., 2013
		8	Unknown	[27] Busch et al., 2013
**I135R**	3	3	BRRS	[44] Boccone et al., 2006
**I135V**	2.7	1	BRRS	[10] Marsh et al., 1999
		3	Unknown	[3] Bubien et al., 2013
		2	Unknown	[3] Bubien et al., 2013
		5	Unknown	[3] Bubien et al., 2013
		3	Unknown	[3] Bubien et al., 2013
		1	Unknown	[3] Bubien et al., 2013
		4	CS	[3] Bubien et al., 2013
**C136R**	5.6	7	CS	[45] Kubo et al., 2000
		7	CS	[3] Bubien et al., 2013
		2	CS	[3] Bubien et al., 2013
		6	CS	[3] Bubien et al., 2013
		6	CS	[6] Ngeow et al., 2014
**R142P**	3	3	CS	[3] Bubien et al., 2013
**A151B**	3	3	CS	[3] Bubien et al., 2013
**Y155N**	4	4	CS	[3] Bubien et al., 2013
**Y155C**	3.66	2	CS	[46] Gicquel et al., 2003
		7	CS	[3] Bubien et al., 2013
		2	CS	[29] Ngeow et al., 2013
**E157G**	1	1	ASD	[16] Varga et al., 2009
**V158I**	3	4	Unknown	[32] De Vivo et al., 2000
		2	Unknown	[32] De Vivo et al., 2000
**D162E**	1	1	CS	[9] Mester et al., 2012
**G165E**	5	5	CS	[47] Nelen et al., 1999
**G165V**	4	4	CS	[20] Marsh et al., 1998
**G165R**	2	2	CS	[48] Banneau et al., 2010
**T167N**	1	1	ASD	[40] O'Roak et al., 2012
**S170R**	4	2	BRRS	[49] Marsh et al., 1997
		7	CS	[3] Bubien et al., 2013
		4	Unknown	[3] Bubien et al., 2013
		3	CS	[3] Bubien et al., 2013
**S170I**	4	4	CS	[6] Ngeow et al., 2014
**Q171E**	0	0	ASD	[2] Vanderver et al., 2014
**R173C**	2.5	1	BRRS	[50] Lachlan et al., 2007
		4	CS	[6] Ngeow et al., 2014
**R173P**	3.5	3	LDD	[51] Kirches et al., 2010
		4	Unknown	[51] Kirches et al., 2010

Mutations	Index score average	Index score patients	Assigned Phenotype	References
**R173H**	0.75	0	Unknown	[50] Lachlan et al., 2007
		2	Unknown	[50] Lachlan et al., 2007
		1	CS/ASD	[3] Bubien et al., 2013
		0	ASD	[33] McBride et al., 2010
**R173G**	2	2	CS	[6] Ngeow et al., 2014
**Y176C**	1	1	ASD	[31] Orrico et al., 2009
**L181P**	1	1	CS	[52] Thiffault et al., 2004
**T202I**	2	2	Unknown	[16] Varga et al., 2009
**C211W**	1	1	ASD	[2] Vanderver et al., 2014
**V217D**	2	2	CS	[53] Kim et al., 2005
**R234Q**	2	2	Multiple cancers	[54] Staal et al., 2002
**F241S**	0	0	ASD	[25] Butler et al., 2005
**P246L**	1	2	BRRS	[10] Marsh et al., 1999
		1	BRRS	[9] Mester et al., 2012
		0	ASD	[2] Vanderver et al., 2014
**D252G**	1	1	ASD	[25] Butler et al., 2005
		1	CS	[3] Bubien et al., 2013
**D252V**	0	0	ASD	[4] Frazier et al., 2014
**V255A**	0	0	ASD	[14] Klein et al., 2013
**W274L**	1	1	DD	[33] McBride et al., 2010
**N276S**	1	1	ASD	[31] Orrico et al., 2009
**T277R**	1.66	1	CS	[3] Bubien et al., 2013
		2	CS	[3] Bubien et al., 2013
		2	CS	[3] Bubien et al., 2013
		3	CS	[3] Bubien et al., 2013
		0	CS/ASD	[3] Bubien et al., 2013
		2	CS	[48] Banneau et al., 2010
**K289E**	1	1	CS	[55] Chi et al., 1998
**D326N**	1	1	AUTISM	[7] Buxbaum et al., 2007
**R335L**	4	4	CS	[56] Sawada et al., 2000
**F337S**	2	2	CS	[50] Lachlan et al., 2007
**K342N**	2	3	HTS	[3] Bubien et al., 2013
		1	CS	[3] Bubien et al., 2013
**V343E**	3.5	4	CS	[57] Lynch et al., 1997
		4	CS	[57] Lynch et al., 1997
		5	CS	[57] Lynch et al., 1997
		3	CS	[57] Lynch et al., 1997
		2	CS	[57] Lynch et al., 1997
		3	CS	[57] Lynch et al., 1997
**L345V**	4.5	4	CS	[6] Ngeow et al., 2014
		5	CS	[27] Busch et al., 2013

ASD, Autism spectrum disorder; BC, Breast cancer; BRRS, Bannayan-Riley-Ruvalcaba Syndrome; CS, Cowden Syndrome; DD, Developmental Delay; HTS, Hamartoma Tumour Syndrome; JPS, Juvenile Polyposis Coli; LDD, Lhermitte-Duclos disease; MR, Mental Retardation; PHTS, PTEN Hamartoma-Tumour Syndrome; PLS, Proteus-Like Syndrome; PS; Proteus Syndrome.

MILD	MODERATE	SEVERE

Table S4 Clinical features of the 204 patients carrying germline-derived PTEN missense mutations.

Mutation	**M1I**	**M1I**	**K6I**	**K6E**	**N12T**	**K13E**	**K13E**	**K13E**	**R14G**	**R15S**
Diagnosis	ASD	ASD	ASD		ASD	CS	CS	CS	ASD	CS
Sex	M	M	M	F	M				F	
Age	11y	12y	3y	11m	5y2m				3y3m	
**CNS**										
Macrocephaly	X	X	X	X	X	X	X		X	X
LDD										
Benign Tumour										
Malignant Tumour										
MR/DD			X	X						
**THYROID**										
Benign (adenoma, goitre)						X	X			
Malignant										X
**BREASTS**										
Benign										
Malignant Tumour										
**SKIN/MUCOSA**										
Trichilemmona										
MCL, FP, AK, F	X	X				X	X	X		
OP	X	X				X	X	X		
SP								X		
Lipomas								X		
Malignant Tumour										
**GASTROINTESTINAL TRACT**										
GI polyps						X				
Benign (hamartoma)										X
Malignant Tumour										
**OTHER BENIGN**						GU les				
**OTHER CANCERS**							LUNG			
**SI SCORE**	1	1	1	1	0	4	4	1	0	3
**REFERENCES**	Hobert et al., 2014	Hobert et al., 2014	Vanderver et al., 2014	Vanderver et al., 2014	Frazier et al., 2014	Bubien et al., 2013	Bubien et al., 2013	Bubien et al., 2013	Frazier et al., 2014	Nagy et al., 2011

Mutation	**Y16H**	**D22E**	**D24Y**	**D24Y**	**D24G**	**D24H**	**D24V**	**L25F**	**T26P**	**Y27N**
Diagnosis	CS	ASD	BRRS	CS	CS	CS	CS	ASD		CS
Sex	F	M	M		F	F	F	M	F	F
Age	65y		4y		49y	46y	28y	7y	19y	46y
**CNS**										
Macrocephaly		X	X				X	X	X	
LDD									X	
Benign Tumour										
Malignant Tumour										
MR/DD			X							
**THYROID**										
Benign (adenoma, goitre)				X			X		X	
Malignant										
**BREASTS**										
Benign				X						
Malignant Tumour	X			X	X	X	X			X
**SKIN/MUCOSA**										
Trichilemmona										
MCL, FP, AK, PPK, F				X			X			
OP				X						
SP			X							
Lipomas			X							
Malignant Tumour				X						
**GASTROINTESTINAL TRACT**										
GI polyps										
Benign (hamartoma)										
Malignant Tumour										
**OTHER BENIGN**				GU les			UTLM			
**OTHER CANCERS**	ENCA				ENCA	ENCA-RC				RC
**SI SCORE**	4	0	2	8	4	6	5	0	2	4
**REFERENCES**	Ngeow et al., 2014	Buxbaum et al., 2007	Celebi et al., 2009	Bubien et al., 2013	Ngeow et al., 2014	Ngeow et al., 2014	Ngeow et al., 2014	Mester et al., 2012	Mester et al., 2012	Ngeow et al., 2014

Mutation	**Y27C**	**P30L**	**A34D**	**A34D**	**M35R**	**M35T**	**G36R**	**P38H**	**A39P**	**G44D**
Diagnosis		CS	BRRS	BRRS	JPC	PLS	CS	ASD	CS	
Sex	F		M	M	M	M	F	M	F	F
Age	3y10m			10y	10y	7y	47y	15y	50-59y	27m
**CNS**										
Macrocephaly		X		X		X	X	X	X	X
LDD	X									
Benign Tumour										
Malignant Tumour										
MR/DD	X			X		X				X
**THYROID**										
Benign (adenoma, goitre)									X	
Malignant										
**BREASTS**										
Benign									FBD	
Malignant Tumour							X		X	
**SKIN/MUCOSA**										
Trichilemmona							X			
MCL, FP, AK, PPK, F		X					X		X	
OP							X		X	
SP		X	X	X		X		X		
Lipomas			X	X		X		X		
Malignant Tumour										
**GASTROINTESTINAL TRACT**										
GI polyps						X	X		X	
Benign (hamartoma)					JPC					
Malignant Tumour										
**OTHER BENIGN**			VASC les			RH,NP,HN	UF		UTLM	Hreflux
**OTHER CANCERS**										
**SI SCORE**	1	1	2	2	1	4	5	1	7	2
**REFERENCES**	Vanderver et al., 2014	Bubien et al., 2013	Marsh et al., 1999	Marsh et al., 1999	Olschwang et al., 1998	Zhou et al., 2001	Celebi et al., 2000	Klein et al., 2013	Tate et al., 2008	Varga et al., 2009

Mutation	N48K	I50T	H61D	H61R	Y65C	Y65C	I67R	Y68N	Y68D	Y68D	Y68H
Diagnosis	CS		VATER	CS	ASD	ASD	CS	ASD	PS	CS	BRRS
Sex	F	M	M	F	M	F			M	F	M
Age	38y	8m	18m	47y	4y5m	11m		4y	3y		
**CNS**											
Macrocephaly		X	X		X	X		X	X	X	
LDD											
Benign Tumour											
Malignant Tumour											
MR/DD		X									
**THYROID**											
Benign (adenoma, goitre)	X										
Malignant											
**BREASTS**											
Benign							X				
Malignant Tumour	X			X			X				
**SKIN/MUCOSA**											
Trichilemmona	X										
MCL, FP, AK, PPK, F	X						X				
OP	X						X				
SP								X			X
Lipomas							X	X	X		X
Malignant Tumour											
**GASTROINTESTINAL TRACT**											
GI polyps	X						X	X			
Benign (hamartoma)											
Malignant Tumour											
**OTHER BENIGN**	OVC										
**OTHER CANCERS**	ENCA			CRC			ENCA				
**SI SCORE**	8	1	0	4	0	0	7	2	1	0	1
**REFERENCES**	Vega et al., 2003	Vanderver et al., 2014	Reardon et al., 2001	Ngeow et al., 2014	Vanderver et al., 2014	Vanderver et al., 2014	Bubien et al., 2013	Klein et al., 2013	Loffeld et al., 2006	Loffeld et al., 2006	Marsh et al., 2008

Mutation	**Y68H**	**Y68H**	**Y68C**	**L70P**	**L70V**	**T78A**	**A79T**	**N82T**	**Y88C**	**D92A**
Diagnosis	CS	ASD	CS	CS	ASD	ASD	BC	BC	PHTS	CS
Sex	F	M	F	M	M		F	F	M	
Age	75y	2y	46y	31y	13y		36y	35y	1y	
**CNS**										
Macrocephaly		X			X	X			X	X
LDD										
Benign Tumour										
Malignant Tumour										
MR/DD					X				X	
**THYROID**										
Benign (adenoma, goitre)	X				X					X
Malignant			X	X						
**BREASTS**										
Benign	X						X	X		X
Malignant Tumour			X				X	X		
**SKIN/MUCOSA**										
Trichilemmona	X									
MCL, FP, AK, PPK, F	X									X
OP	X				X					X
SP										
Lipomas					X					X
Malignant Tumour										
**GASTROINTESTINAL TRACT**										
GI polyps										
Benign (hamartoma)										
Malignant Tumour										
**OTHER BENIGN**										
**OTHER CANCERS**										OVCA
**SI SCORE**	3	0	4	2	3	0	3	3	1	5
**REFERENCES**	Marsh et al., 1998	Frazier et al., 2014	Ngeow et al., 2014	Marsh et al., 1998	Hobert et al., 2014	Schaaf et al., 2011	Figer et al., 2002	Figer et al., 2002	Mester et al., 2012	Bubien et al., 2013

Mutation	H93Y	H93Y	H93Y	H93R	P96Q	P96Q	P96R	P96R	C105Y	D107G
Diagnosis	CS	CS	ASD	ASD	CS	CS	CS	CS	BRRS	
Sex	M	F	M	M	M	F	F	F		M
Age	46y	48y	4y4m	4y	72y	42y	30y	26y		3y
**CNS**										
Macrocephaly	X		X	X			X	X		X
LDD										
Benign Tumour										
Malignant Tumour										
MR/DD				X						X
**THYROID**										
Benign (adenoma, goitre)	X				X	X	X	X		
Malignant										
**BREASTS**										
Benign							X			
Malignant Tumour		X								
**SKIN/MUCOSA**										
Trichilemmona	X				X		X			
MCL, FP, AK, PPK, F					X	X				
OP					X		X			
SP									X	
Lipomas							X	X	X	
Malignant Tumour										
**GASTROINTESTINAL TRACT**										
GI polyps	X				X			X	X	
Benign (hamartoma)										
Malignant Tumour										
**OTHER BENIGN**	VASC les									
**OTHER CANCERS**		ENCA					VASC les			
**SI SCORE**	4	4	0	1	3	2	4	3	2	1
**REFERENCES**	Kohno et al., 1998	Ngeow et al., 2014	Frazier et al., 2014	Butler et al., 2005	Bussaglia et al., 2002	Bussaglia et al., 2002	Busch et al., 2013	Mester et al., 2012	Marsh et al., 1999	Vanderver et al., 2014

Mutation	L108P	L108P	L108P	L112P	H118P	V119I	V119I	H123Q	H123Q	H123R
Diagnosis	PHTS	PHTS	CS	CS	ASD	Mcancer	Mcancer	ASD	CS	CS
Sex			F	F		F	F	F	F	M
Age			50y	26y				2y6m	28y	
**CNS**										
Macrocephaly	X			X	X			X	X	X
LDD				X						
Benign Tumour										
Malignant Tumour				X						
MR/DD	X			X	X			X		
**THYROID**										
Benign (adenoma, goitre)		X	X	X						X
Malignant										X
**BREASTS**										
Benign	FBD	X	X	X					X	
Malignant Tumour		X	X			X	X			
**SKIN/MUCOSA**										
Trichilemmona				X						
MCL, FP, AK, PPK, F				X						X
OP				X						
SP										
Lipomas	X	X								
Malignant Tumour				X						
**GASTROINTESTINAL TRACT**										
GI polyps		X								
Benign (hamartoma)										
Malignant Tumour			X						X	
**OTHER BENIGN**	VASC les	GU les							H of leg	
**OTHER CANCERS**	OVCA		ENCA	ENCA		LUNG	ENCA			
**SI SCORE**	6	7	7	11	1	4	4	1	4	4
**REFERENCES**	Tan et al., 2007	Bubien et al., 2013	Ngeow et al., 2013	Tsou et al., 1998	Orrico et al., 2009	deVivo et al., 2000	de Vivo et al., 2000	McBride et al., 2010	Kersseboom et al., 2012	Nelen et al., 1997

Mutation	**H123D**	**C124R**	**C124R**	**C124R**	**A126P**	**K128E**	**K128N**	**G129R**	**G129R**	**G129E**
Diagnosis	CS	CS	CS	CS	CS	CS	CS	CS		CS
Sex	F	F			M	M	M	F	M	
Age	53y				35y	23y	35y	14y7m		
**CNS**										
Macrocephaly		X	X		X	X		X	X	
LDD										
Benign Tumour						X		X		
Malignant Tumour								X		
MR/DD								X		
**THYROID**										
Benign (adenoma, goitre)	X	X	X	X	X	X			X	X
Malignant										
**BREASTS**										
Benign			X	X						X
Malignant Tumour										
**SKIN/MUCOSA**										
Trichilemmona	X									
MCL, FP, AK, PPK, F	X	X	X	X						X
OP	X					X				
SP					X					
Lipomas					X	X				
Malignant Tumour										
**GASTROINTESTINAL TRACT**										
GI polyps								X		
Benign (hamartoma)			X	X	X		X			X
Malignant Tumour				X			X			
**OTHER BENIGN**	OC					VASC les			VASC les	
**OTHER CANCERS**										
**SI SCORE**	3	2	4	6	3	4	3	5	2	4
**REFERENCES**	Bussaglia et al., 2002	Nelen et al., 1997	Nelen et al., 1997	Nelen et al., 1997	Mester et al., 2012	Busch et al., 2013	Ngeow et al., 2013	Elia et al, 2012	UNPUB	Liaw et al., 1997

Mutation	G129E	G129E	G129E	G129E	R130Q	R130Q	R130Q	R130Q	R130Q	R130Q
Diagnosis	CS	CS	CS	CS	CS	CS/BRRS	CS/BRRS	CS/BRRS	CS	CS
Sex		M	M	F	M	M	M	M	F	M
Age				60y	35y	23y	25y	22y	48y	17y
**CNS**										
Macrocephaly				X		X	X	X	X	X
LDD										
Benign Tumour										
Malignant Tumour		X								
MR/DD						X	X			
**THYROID**										
Benign (adenoma, goitre)			X	X	X	X		X		X
Malignant										
**BREASTS**										
Benign									X	
Malignant Tumour	X	X							X	
**SKIN/MUCOSA**										
Trichilemmona		X	X							
MCL, FP, AK, PPK, F	X				X	X	X			X
OP						X	X			
SP						X	X	X		X
Lipomas				X			X			X
Malignant Tumour	X	X								
**GASTROINTESTINAL TRACT**										
GI polyps					X			X		
Benign (hamartoma)						X	X	X	X	X
Malignant Tumour								X		
**OTHER BENIGN**									UF	LPH
**OTHER CANCERS**					LUNG					
**SI SCORE**	5	7	2	2	5	4	3	3	5	4
**REFERENCES**	Liaw et al., 1997	Liaw et al., 1997	Liaw et al., 1997	Ngeow et al., 2013	Kurose et al., 1999	Kurose et al., 1999	Kurose et al., 1999	Kurose et al., 1999	Heindl et al., 2012	Heindl et al., 2012

Mutation	R130Q	R130Q	R130Q	R130G	R130G	R130G	R130G	R130G	R130G	R130L
Diagnosis	CS	CS	CS	CS		CS	CS		CS	CS
Sex	F	F	F	F	F					
Age	42y	29y	48y		40y					
**CNS**										
Macrocephaly	X				X	X	X	X	X	X
LDD										
Benign Tumour										
Malignant Tumour										
MR/DD										
**THYROID**										
Benign (adenoma, goitre)	X				X				X	X
Malignant										
**BREASTS**										
Benign	X									
Malignant Tumour	X	X	X	X	X					
**SKIN/MUCOSA**										
Trichilemmona										
MCL, FP, AK, PPK, F						X		X	X	X
OP						X			X	X
SP									X	X
Lipomas	X						X	X	X	
Malignant Tumour										
**GASTROINTESTINAL TRACT**										
GI polyps										X
Benign (hamartoma)										
Malignant Tumour										
**OTHER BENIGN**					UF					
**OTHER CANCERS**		OVCA								
**SI SCORE**	5	4	2	2	4	1	1	1	2	3
**REFERENCES**	Ngeow et al., 2013	Ngeow et al., 2014	Ngeow et al., 2014	Lobo et al., 2009	Mester et al., 2012	Bubien et al., 2013	Bubien et al., 2013	Bubien et al., 2013	Bubien et al., 2013	Bubien et al., 2013

Mutation	**R130L**	**R130L**	**R130L**	**T131I**	**T131I**	**T131I**	**G132A**	**G132D**	**G132D**	**G132D**
Diagnosis	CS	CS	CS	CS/ASD		ASD	PHTS	CS	CS	CS
Sex			F			F		M	M	
Age			21y			4y1m		27y	53y	
**CNS**										
Macrocephaly		X		X	X	X	X		X	X
LDD										
Benign Tumour										
Malignant Tumour								DGCC		
MR/DD				X		X	X			
**THYROID**										
Benign (adenoma, goitre)	X	X			X				X	
Malignant		X	X							X
**BREASTS**										
Benign		X			X					X
Malignant Tumour		X								X
**SKIN/MUCOSA**										
Trichilemmona										
MCL, FP, AK, PPK, F	X	X							X	X
OP	X								X	X
SP	X									
Lipomas										
Malignant Tumour										
**GASTROINTESTINAL TRACT**										
GI polyps	X	X			X					
Benign (hamartoma)										
Malignant Tumour		X								
**OTHER BENIGN**										
**OTHER CANCERS**			UBC						RC	
**SI SCORE**	3	10	4	1	3	1	1	2	4	6
**REFERENCES**	Bubien et al., 2013	Marsh et al., 1998	Ngeow et al., 2014	Bubien et al., 2013	Bubien et al., 2013	O'Roak et al., 2012	Tan et al., 2007	Derrey et al., 2004	Heindl et al., 2012	Bubien et al., 2013

Mutation	**G132D**	**G132D**	**G132D**	**G132V**	**M134R**	**M134R**	**M134R**	**M134R**	**M134I**	**M134I**
Diagnosis	CS	CS	ASD	PHTS	BRRS	BRRS	BRRS			
Sex			M	M	M	M	M	F	F	M
Age			4y	4y6m	18y	5y		46y	38y	17y
**CNS**										
Macrocephaly	X	X	X	X	X	X	X	X	X	X
LDD	X									
Benign Tumour										
Malignant Tumour										
MR/DD				X						
**THYROID**										
Benign (adenoma, goitre)	X	X								
Malignant										
**BREASTS**										
Benign	X	X								
Malignant Tumour								X		
**SKIN/MUCOSA**										
Trichilemmona										
MCL, FP, AK, PPK, F		X								
OP		X								
SP					X	X	X			
Lipomas	X			X						
Malignant Tumour										
**GASTROINTESTINAL TRACT**										
GI polyps				X				X		
Benign (hamartoma)										
Malignant Tumour										
**OTHER BENIGN**										
**OTHER CANCERS**										
**SI SCORE**	4	3	0	3	0	0	0	3	0	0
**REFERENCES**	Bubien et al 2013	Bubien et al., 2013	Frazier et al., 2014	Tekin et al., 2006	Figer et al., 2002	Figer et al., 2002	Figer et al., 2002	Mester et al., 2012	Busa et al., 2013	Busa et al., 2013

Mutation	**M134I**	**M134I**	**M134T**	**M134T**	**M134T**	**I135R**	**I135V**	**I135V**	**I135V**	**I135V**
Diagnosis			MR	CS		BRRS	BRRS			
Sex	F	F	F	F	F					
Age	15y	8y	9y3m	44y	46y					
**CNS**										
Macrocephaly	X	X		X	X	X		X	X	X
LDD										
Benign Tumour										
Malignant Tumour										
MR/DD			X			X			X	
**THYROID**										
Benign (adenoma, goitre)				X	X			X		X
Malignant					X					
**BREASTS**										
Benign				X	X			X		X
Malignant Tumour				X	X					
**SKIN/MUCOSA**										
Trichilemmona					X					
MCL, FP, AK, PPK, F					X			X	X	X
OP				X	X			X	X	X
SP							X			
Lipomas							X			X
Malignant Tumour										
**GASTROINTESTINAL TRACT**										
GI polyps						X				X
Benign (hamartoma)										
Malignant Tumour										
**OTHER BENIGN**					VASC les	LH				GU les
**OTHER CANCERS**										
**SI SCORE**	0	0	1	5	8	3	1	3	2	5
**REFERENCES**	Busa et al., 2013	Busa et al., 2013	McBride et al., 2010	Ngeow et al., 2013	Busch et al., 2013	Boccone et al., 2006	Marsh et al., 2009	Bubien et al., 2013	Bibien et al., 2013	Bubien et al., 2013

Mutation	I135V	I135V	I135V	C136R	C136R	C136R	C136R	C136R	R142P	A151D
Diagnosis		ASD	CS	CS	CS	CS/MR/AS D	CS	CS	CS	CS
Sex				F				F		
Age				30y				33y		
**CNS**										
Macrocephaly	X	X	X			X			X	X
LDD							X			
Benign Tumour										
Malignant Tumour										
MR/DD						X				
**THYROID**										
Benign (adenoma, goitre)	X		X	X			X		X	X
Malignant					X		X	X		
**BREASTS**										
Benign			X	X	X					
Malignant Tumour					X			X		
**SKIN/MUCOSA**										
Trichilemmona										
MCL, FP, AK, PPK, F	X		X	X	X	X	X		X	X
OP	X		X	X			X		X	X
SP							X			
Lipomas		X	X			X				X
Malignant Tumour										
**GASTROINTESTINAL TRACT**										
GI polyps	X			X	X		X		X	X
Benign (hamartoma)										
Malignant Tumour				X						
**OTHER BENIGN**			GU les	OC						
**OTHER CANCERS**								ENCA		
**SI SCORE**	3	1	4	7	7	2	6	6	3	3
**REFERENCES**	Bubien et al., 2013	Bubien et al., 2013	Bubien et al., 2013	Kubo et al., 2000	Bubien et al., 2013	Bubien et al., 2013	Bubien et al., 2013	Ngeow et al., 2014	Bubien et al., 2013	Bubien et al., 2013

Mutation	**Y155N**	**Y155C**	**Y155C**	**Y155C**	**E157G**	**V158I**	**V158I**	**D162E**	**G165R**	**G165E**
Diagnosis	CS	CS	CS	CS	ASD			CS	CS	CS
Sex		F		F		F	F	M		F
Age		50y		66y	3y8m			3y	59y	
**CNS**										
Macrocephaly			X	X	X			X		X
LDD										
Benign Tumour										
Malignant Tumour										
MR/DD					X			X		
**THYROID**										
Benign (adenoma, goitre)	X		X							X
Malignant										X
**BREASTS**										
Benign			X	X						X
Malignant Tumour			X			X			X	
**SKIN/MUCOSA**										
Trichilemmona										
MCL, FP, AK, PPK, F	X		X							X
OP	X		X							
SP										
Lipomas			X	X						
Malignant Tumour	X									
**GASTROINTESTINAL TRACT**										
GI polyps			X							
Benign (hamartoma)										
Malignant Tumour										
**OTHER BENIGN**			GU les							
**OTHER CANCERS**		MT				LGTC	L			
**SI SCORE**	4	2	7	2	1	4	2	1	2	5
**REFERENCES**	Bubien et al., 2013	Gicquel et al., 2003	Bubien et al., 2013	Ngeow et al., 2013	Varga et al., 2009	de Vivo et al., 2000	de Vivo et al., 2000	Mester et al., 2012	Bannean et al., 2010	Nelen et al., 1999

Mutation	G165V	T167N	S170R	S170R	S170R	S170R	S170I	Q171E	R173C	R173C
Diagnosis	CS	ASD	BRRS	CS		CS	CS	ASD	BRRS	CS
Sex		F					F	F		F
Age		8y2m					17y	3y10m		54y
**CNS**										
Macrocephaly	X	X	X	X	X	X		X	X	
LDD						X				
Benign Tumour	X									
Malignant Tumour										
MR/DD		X							X	
**THYROID**										
Benign (adenoma, goitre)	X				X	X				
Malignant				X	X		X			X
**BREASTS**										
Benign	X			X						
Malignant Tumour				X						X
**SKIN/MUCOSA**										
Trichilemmona										
MCL, FP, AK, PPK, F	X				X	X				
OP					X	X				
SP			X						X	
Lipomas			X	X	X	X				
Malignant Tumour										
**GASTROINTESTINAL TRACT**										
GI polyps				X						
Benign (hamartoma)										
Malignant Tumour										
**OTHER BENIGN**			VASC les- LM							
**OTHER CANCERS**							ENCA			
**SI SCORE**	4	1	2	7	4	3	4	0	1	4
**REFERENCES**	Marsh et al., 1998	O'Roak et al., 2012	Marsh et al., 1997	Bubien et al., 2013	Bubien et al., 2013	Bubien et al., 2013	Ngeow et al., 2014	Vanderver et al., 2014	Lachlan et al., 2007	Ngeow et al., 2014

Mutation	**R173G**	**R173P**	**R173P**	**R173H**	**R173H**	**R173H**	**R173H**	**Y176C**	**L181P**	**T202I**
Diagnosis	CS	LD		CS			ASD	ASD	CS	
Sex	F	F	F		M	M	M			M
Age	44y		42y		9y	43y	4y5m			1y8m
**CNS**										
Macrocephaly			X	X	X	X	X	X	X	
LDD		X	X							
Benign Tumour										
Malignant Tumour										
MR/DD			X	X				X		X
**THYROID**										
Benign (adenoma, goitre)		X	X							
Malignant										
**BREASTS**										
Benign										
Malignant Tumour	X									
**SKIN/MUCOSA**										
Trichilemmona										
MCL, FP, AK, PPK, F		X	X							X
OP						X				
SP						X				
Lipomas										
Malignant Tumour										
**GASTROINTESTINAL TRACT**										
GI polyps						X				
Benign (hamartoma)										
Malignant Tumour										
**OTHER BENIGN**									VASC les	
**OTHER CANCERS**										
**SI SCORE**	2	3	4	1	0	2	0	1	1	2
**REFERENCES**	Ngeow et al., 2014	Kirches et al., 2010	Kirches et al., 2010	Bubien et al., 2013	Lachlan et al., 2007	Lachlan et al., 2007	McBride et al., 2010	Orrico et al., 2009	Thiffault et al., 2004	Varga et al., 2009

Mutation	**C211W**	**V217D**	**R234Q**	**F241S**	**P246L**	**P246L**	**P246L**	**D252V**	**D252G**	**D252G**
Diagnosis	ASD	CS	Mcancer	ASD	BRRS	BRRS	ASD	ASD	ASD	CS
Sex	M		M	M		M	M	F	M	
Age	3y10m		38y	2y5m		2y	5y	15y5m	3y5m	
**CNS**										
Macrocephaly	X					X	X	X	X	X
LDD										
Benign Tumour										
Malignant Tumour			OD, MnG							
MR/DD	X					X			X	
**THYROID**										
Benign (adenoma, goitre)										
Malignant										
**BREASTS**										
Benign										
Malignant Tumour										
**SKIN/MUCOSA**										
Trichilemmona										
MCL, FP, AK, PPK, F										X
OP		X								X
SP				X						
Lipomas					X					
Malignant Tumour										
**GASTROINTESTINAL TRACT**										
GI polyps		X								
Benign (hamartoma)										
Malignant Tumour										
**OTHER BENIGN**					VASC les					
**OTHER CANCERS**										
**SI SCORE**	1	2	2	0	2	1	0	0	1	1
**REFERENCES**	Vanderver et al., 2014	Kim et al., 2005	Staal et al., 2002	Butler et al., 2005	Marsh et al., 1999	Mester et al., 2012	Vanderver et al., 2014	Frazier et al., 2014	Butler et al., 2005	Bubien et al., 2013

Mutation	**V255A**	**W274L**	**N276S**	**T277R**	**T277R**	**T277R**	**T277R**	**T277R**	**T277R**	**K289E**
Diagnosis	ASD	DD	ASD	CS	CS	CS	CS	CS/ASD	CS	CS
Sex	M	F							F	
Age	4y4m	8m							27y	
**CNS**										
Macrocephaly	X	X	X	X	X	X	X	X		
LDD										
Benign Tumour										
Malignant Tumour										
MR/DD		X	X		X					
**THYROID**										
Benign (adenoma, goitre)						X	X			
Malignant										
**BREASTS**										
Benign							X			
Malignant Tumour									X	
**SKIN/MUCOSA**										
Trichilemmona										
MCL, FP, AK, PPK, F				X	X	X	X			
OP				X	X	X	X			
SP										
Lipomas				X			X			
Malignant Tumour										
**GASTROINTESTINAL TRACT**										
GI polyps										X
Benign (hamartoma)										X
Malignant Tumour										
**OTHER BENIGN**										
**OTHER CANCERS**										
**SI SCORE**	0	1	1	1	2	2	3	0	2	1
**REFERENCES**	Klein et al., 2013	McBride et al., 2010	Orrico et al., 2009	Bubien et al., 2013	Bubien et al., 2013	Bubien et al., 2013	Bubien et al., 2013	Bubien et al., 2013	Banneau et al., 2010	Chi et al., 1998

Mutation	D326N	R335L	F337S	K342N	K342N	V343E	V343E	V343E	V343E	V343E	V343E	L345V	L345V
Diagnosis	ASD	CS	CS	HTS	CS	CS	CS	CS	CS	CS	CS	CS	CS
Sex		F	M			F	F	F	M	F	F	F	F
Age		48y	9y									36y	58y
**CNS**													
Macrocephaly	X		X	X	X								
LDD													
Benign Tumour													
Malignant Tumour													
MR/DD	X												
**THYROID**													
Benign (adenoma, goitre)		X				X	X	X					X
Malignant				X							X		
**BREASTS**													
Benign							X	X			X		X
Malignant Tumour		X				X				X		X	X
**SKIN/MUCOSA**													
Trichilemmona		X											
MCL, FP, AK, PPK, F		X					X						X
OP		X	X	X									X
SP			X	X									
Lipomas			X	X	X								
Malignant Tumour													
**GASTROINTESTINAL TRACT**													
GI polyps			X				X	X	X				
Benign (hamartoma)													
Malignant Tumour													
**OTHER BENIGN**						NE							
**OTHER CANCERS**								ENCA	PGT			ENCA	
**SI SCORE**	1	4	2	3	1	4	4	5	3	2	3	4	5
**REFERENCES**	Buxbaum et al., 2007	Sawada et al., 2000	Lachlan et al., 2007	Bubien et al., 2013	Bubien et al., 2013	Lynch et al., 1997	Lynch et al., 1997	Lynch et al., 1997	Lynch et al., 1997	Lynch et al., 1997	Lynch et al., 1997	Ngeow et al., 2014	Busch et al., 2013

Abbreviations

AK	Acral Keratosis
CNS	Central Nervous System
CRC	Colorectal Cancer
DGCC	Dysplastic Gangliocytoma of the Cerebellum
ENCA	Endometrial Cancer
F	Fibromas
FBD	Fibrocystic disease
FP	Facial papules
GgNa	Ganglioneuroma
GI	Gastointestinal
GU les	Genitourinary lesions
H	Hemangioma
H reflux	Hernia reflux
HN	Hydronephrosis
JPC	Juvenile Polyposis Coli
L	Lymphoma
LDD	Lhermitte-Duclos disease
LGTC	Lower Genital Tract Cancer
LH	Lymphoid Hyperplasia
LM	Leiomyoma
LPH	Lipomatous Hemangioma
MCL	Mucocutaneus Lesions
MnG	Meningioma
MR/DD	Mental Retardation/Developmental Delay
MT	Multiple Tumours
NE	Neuroma
NP	Nephrolithiasis
OC	Ovaric Cysts
OD	Oligodendrioma
OP	Oral mucosal Papillomatosis
OVCA	Ovarian cancer
PGT	Papillary Carcinoma
PLS	Proteus-Like Syndrome
PPK	Palmoplantar Keratoses
RC	Renal Carcinoma
RH	Renal Hamartoma
SP	Speckled Penis
UBC	Urinary Bladder Cancer
UF	Uterin Fibroids
UNPUB	Unplublished
UTLM	Uterine Leiomyoma
VASC les	Vascular lesions

Supporting Materials and Methods

Expression vectors and recombinant PTEN protein expression

Plasmid and lentiviral expression vectors for untagged and glutathione S-transferase (GST)-tagged human PTEN have been previously described [58 59]. The mutant expression vectors derived from pGEX6P1 PTEN and pHR-SIN PTEN were prepared by site-directed mutagenesis of the corresponding PTEN WT constructs with primers containing missense mutations. Recombinant wild-type and mutant forms of PTEN were expressed in Escherichia coli and purified by glutathione-affinity chromatography.

Immunoblotting

Protein gel electrophoresis was conducted with 10 µg of total soluble protein per sample using NuPage Bis-Tris 4-12% gradient polyacrylamide gels (Invitrogen Life Technologies) following manufacturers protocols. Proteins were transferred onto PVDF membrane (Perkin-Elmer) and membranes blocked in 5% milk powder/TBST for 1 hour at room temperature (RT). Blocked membranes were incubated overnight with primary antibodies. Antibodies used were: anti-P(Ser473)Akt and anti-P(Thr308)Akt (Cat. n.9271L and 9275L, Cell Signaling Technology), anti-PTEN (A2B1 Cat. n. sc-7974, Santa Cruz Biotechnology and PTEN 138G6, Cat. n. 9559S, Cell Signaling Technology), anti-GAPDH (Cat. n. AB2302, Millipore), anti-Akt (Cat. n. 9272, Cell Signaling Technology), anti-P(Thr246)PRAS40 (Cat. n. 29975, Cell Signaling Technology), anti-PRAS40 (Cat. n. 2691S, Cell Signaling Technology). Antibody complexes were detected by 1 hour incubation at RT with HRP conjugated secondary antibodies (Vector Labs). Blots were developed with ECL plus (Millipore)

and chemiluminescence imaged directly using an ImageQuant LAS4000 imaging system.

Lipid phosphatase assay

The preparation of 3-^{33}P labelled phosphoinositide substrate has been described previously ([58 59]. To test the lipid phosphatase activity of PTEN against PtdIns(3,4,5)P_3, lipid vesicles were prepared by sonication containing 100 μM phosphatidylcholine (PC), 1 μM unlabelled diC16 PtdIns(3,4,5)P_3 and a volume of ^{33}P-PtdIns(3,4,5)P_3 to give 100,000 cpm per assay. PTEN was immunoprecipitated using anti-PTEN A2B1 antibody in lysis buffer (50 mM Tris-HCL pH 7.4, 150 mM NaCl, 1 mM EDTA, 1 mM EGTA, 5 mM Sodium pyrophosphate, 10 mM β-glycerophosphate, 50 mM Sodium fluoride, 1% NP-40 and protease inhibitors) and washed twice with high salt (300 mM NaCl) lysis buffer and then with reaction buffer (50 mM Hepes pH 7.4, 1 mM EGTA, 10 mM dithiothreitol (DTT) and 150 mM NaCl). Substrate vesicles were incubated in the reaction buffer with PTEN immune complexes or with 500 ng of recombinant PTEN protein. After 1 hour of incubation at 37ºC, the reactions were stopped through the addition of 10 μl of Bovine Serum Albumin (essentially fatty acid free- Roche) (10 mg/ml) and 500 μl of 1M ice cold perchloric acid (PCA). The samples were mixed and incubated for 30 minutes on ice, followed by centrifugation at 14,000 rpm for 10 minutes at 4ºC to remove lipid and protein. 10% (w/v) ammonium molybdate was added to the supernatant to allow the inorganic phosphate to partition into the organic phase. After incubation of 10 minutes at room temperature, 1 ml Toluene:Isobutyl alcohol (1:1 v/v) was added. A two-phase mix was formed, and the upper organic phase, containing the phosphate complex, was then removed, mixed to liquid scintillation cocktail (Scint Safe 3, Fisher Chemical) and radioactivity was counted using a Beckman scintillation counter.

Reverse Transcriptase quantitative PCR (qPCR)

Total cellular RNA was isolated from U87MG cells expressing PTEN WT and mutants by using TRIzol (Life Technologies - Invitrogen) and RNeasy Mini kit (Qiagen), reversed transcribed into random primed cDNA (RNA to cDNA EcoDry Premix – Random Hexamers Clontech) and used as a template in quantitative PCR reactions to measure PTEN mRNA levels. GAPDH was used as internal control and Power Sybr green as the fluorescent reporter (Applied Biosystems). All reactions were performed according to the manufacturer's instructions on a Step One Plus real time PCR instrument (Life Technologies).

Primers were designed to be able to measure PTEN mRNA levels only and not its pseudogene. PTEN Primers: F: 5'-CCCGGGGGGATCCACTAG; R: 5'-GGATAAATATAGGTCAAG. GAPDH Primers: F: 5'-GTGAAGGTCGGAGTCAACGG; R: 5'-GAGGGATCTCGCTCCTGGAA.

Immunocytochemistry

Neuronal cultures treated as described above were fixed using 4% paraformaldehyde for 30 minutes and then blocked with 3% BSA and 0.1% Triton x100 for 1 hour. Fixed neurons were incubated with anti-GFP antibody (1:1,000, Abcam), anti-PTEN antibody (1:500, PTEN138G6 Cell Signaling Technology) or anti-RFP (1:1,000, Cat. n. ab62341 Abcam) antibody diluted in 3% BSA, 1% goat serum and 1% sodium azide followed by incubation with Alexa488, Alexa568 and Alexa647 conjugated secondary antibodies (1:1,000, Invitrogen). Cells were stained with DAPI (1 μg/ml) for 10 minutes prior to mounting. The soma of the neurons, density and length of the spines were measured using ImageJ and were represented in μm.

Animal procedures

All animal procedures (breeding and sacrifice) were conducted in accordance with local ethical guidelines and approved animal care protocols (Berlin: Institutional Animal Care and Use Committee (IACUC) and the Landesamt für Gesundheit und Soziales (LAGeSO) – license T 0347/11.

Supplemental References

1. Hobert JA, Embacher R, Mester JL, et al. Biochemical screening and PTEN mutation analysis in individuals with autism spectrum disorders and macrocephaly. European journal of human genetics : EJHG 2014;**22**(2):273-6 doi: 10.1038/ejhg.2013.114[published Online First: Epub Date]|.
2. Vanderver A, Tonduti D, Kahn I, et al. Characteristic brain magnetic resonance imaging pattern in patients with macrocephaly and PTEN mutations. American journal of medical genetics Part A 2014;**164A**(3):627-33 doi: 10.1002/ajmg.a.36309[published Online First: Epub Date]|.
3. Bubien V, Bonnet F, Brouste V, et al. High cumulative risks of cancer in patients with PTEN hamartoma tumour syndrome. J Med Genet 2013;**50**(4):255-63 doi: 10.1136/jmedgenet-2012-101339[published Online First: Epub Date]|.
4. Frazier TW, Embacher R, Tilot AK, et al. Molecular and phenotypic abnormalities in individuals with germline heterozygous PTEN mutations and autism. Molecular psychiatry 2014 doi: 10.1038/mp.2014.125[published Online First: Epub Date]|.
5. Nagy R, Ganapathi S, Comeras I, et al. Frequency of germline PTEN mutations in differentiated thyroid cancer. Thyroid 2011;**21**(5):505-10 doi: 10.1089/thy.2010.0365[published Online First: Epub Date]|.
6. Ngeow J, Stanuch K, Mester JL, et al. Second malignant neoplasms in patients with Cowden syndrome with underlying germline PTEN mutations. Journal of clinical oncology : official journal of the American Society of Clinical Oncology 2014;**32**(17):1818-24 doi: 10.1200/JCO.2013.53.6656[published Online First: Epub Date]|.
7. Buxbaum JD, Cai G, Chaste P, et al. Mutation screening of the PTEN gene in patients with autism spectrum disorders and macrocephaly. Am J Med Genet B Neuropsychiatr Genet 2007;**144B**(4):484-91 doi: 10.1002/ajmg.b.30493[published Online First: Epub Date]|.
8. Celebi JT, Tsou HC, Chen FF, et al. Phenotypic findings of Cowden syndrome and Bannayan-Zonana syndrome in a family associated with a single germline mutation in PTEN. J Med Genet 1999;**36**(5):360-4
9. Mester J, Eng C. Estimate of de novo mutation frequency in probands with PTEN hamartoma tumor syndrome. Genetics in medicine : official journal of the American College of Medical Genetics 2012;**14**(9):819-22 doi: 10.1038/gim.2012.51[published Online First: Epub Date]|.
10. Marsh DJ, Kum JB, Lunetta KL, et al. PTEN mutation spectrum and genotype-phenotype correlations in Bannayan-Riley-Ruvalcaba syndrome suggest a single entity with Cowden syndrome. Human molecular genetics 1999;**8**(8):1461-72
11. Olschwang S, Serova-Sinilnikova OM, Lenoir GM, et al. PTEN germ-line mutations in juvenile polyposis coli. Nat Genet 1998;**18**(1):12-4 doi: 10.1038/ng0198-12[published Online First: Epub Date]|.
12. Zhou X, Hampel H, Thiele H, et al. Association of germline mutation in the PTEN tumour suppressor gene and Proteus and Proteus-like syndromes. Lancet 2001;**358**(9277):210-1
13. Celebi JT, Ping XL, Zhang H, et al. Germline PTEN mutations in three families with Cowden syndrome. Exp Dermatol 2000;**9**(2):152-6
14. Klein S, Sharifi-Hannauer P, Martinez-Agosto JA. Macrocephaly as a clinical indicator of genetic subtypes in autism. Autism research : official journal of the International Society for Autism Research 2013;**6**(1):51-6 doi: 10.1002/aur.1266[published Online First: Epub Date]|.
15. Tate G, Suzuki T, Endo Y, et al. A novel mutation of the PTEN gene in a Japanese patient with Cowden syndrome and bilateral breast cancer. Cancer Genet Cytogenet 2008;**184**(1):67-71 doi: 10.1016/j.cancergencyto.2008.03.013[published Online First: Epub Date]|.
16. Varga EA, Pastore M, Prior T, et al. The prevalence of PTEN mutations in a clinical pediatric cohort with autism spectrum disorders, developmental delay, and macrocephaly. Genetics in

medicine : official journal of the American College of Medical Genetics 2009;**11**(2):111-7 doi: 10.1097/GIM.0b013e31818fd762[published Online First: Epub Date]|.

17. Vega A, Torres J, Torres M, et al. A novel loss-of-function mutation (N48K) in the PTEN gene in a Spanish patient with Cowden disease. J Invest Dermatol 2003;**121**(6):1356-9 doi: 10.1111/j.1523-1747.2003.12638.x[published Online First: Epub Date]|.
18. Reardon W, Zhou XP, Eng C. A novel germline mutation of the PTEN gene in a patient with macrocephaly, ventricular dilatation, and features of VATER association. J Med Genet 2001;**38**(12):820-3
19. Loffeld A, McLellan NJ, Cole T, et al. Epidermal naevus in Proteus syndrome showing loss of heterozygosity for an inherited PTEN mutation. Br J Dermatol 2006;**154**(6):1194-8 doi: 10.1111/j.1365-2133.2006.07196.x[published Online First: Epub Date]|.
20. Marsh DJ, Dahia PL, Caron S, et al. Germline PTEN mutations in Cowden syndrome-like families. J Med Genet 1998;**35**(11):881-5
21. Lobo GP, Waite KA, Planchon SM, et al. Germline and somatic cancer-associated mutations in the ATP-binding motifs of PTEN influence its subcellular localization and tumor suppressive function. Human molecular genetics 2009;**18**(15):2851-62 doi: 10.1093/hmg/ddp220[published Online First: Epub Date]|.
22. Schaaf CP, Sabo A, Sakai Y, et al. Oligogenic heterozygosity in individuals with high-functioning autism spectrum disorders. Human molecular genetics 2011;**20**(17):3366-75 doi: 10.1093/hmg/ddr243[published Online First: Epub Date]|.
23. Figer A, Kaplan A, Frydman M, et al. Germline mutations in the PTEN gene in Israeli patients with Bannayan-Riley-Ruvalcaba syndrome and women with familial breast cancer. Clinical genetics 2002;**62**(4):298-302
24. Kohno T, Takahashi M, Fukutomi T, et al. Germline mutations of the PTEN/MMAC1 gene in Japanese patients with Cowden disease. Jpn J Cancer Res 1998;**89**(5):471-4
25. Butler MG, Dasouki MJ, Zhou XP, et al. Subset of individuals with autism spectrum disorders and extreme macrocephaly associated with germline PTEN tumour suppressor gene mutations. J Med Genet 2005;**42**(4):318-21 doi: 10.1136/jmg.2004.024646[published Online First: Epub Date]|.
26. Bussaglia E, Pujol RM, Gil MJ, et al. PTEN mutations in eight Spanish families and one Brazilian family with Cowden syndrome. J Invest Dermatol 2002;**118**(4):639-44 doi: 10.1046/j.1523-1747.2002.01728.x[published Online First: Epub Date]|.
27. Busch RM, Chapin JS, Mester J, et al. Cognitive characteristics of PTEN hamartoma tumor syndromes. Genetics in medicine : official journal of the American College of Medical Genetics 2013;**15**(7):548-53 doi: 10.1038/gim.2013.1[published Online First: Epub Date]|.
28. Tan WH, Baris HN, Burrows PE, et al. The spectrum of vascular anomalies in patients with PTEN mutations: implications for diagnosis and management. J Med Genet 2007;**44**(9):594-602 doi: 10.1136/jmg.2007.048934[published Online First: Epub Date]|.
29. Ngeow J, Heald B, Rybicki LA, et al. Prevalence of germline PTEN, BMPR1A, SMAD4, STK11, and ENG mutations in patients with moderate-load colorectal polyps. Gastroenterology 2013;**144**(7):1402-9, 09 e1-5 doi: 10.1053/j.gastro.2013.02.001[published Online First: Epub Date]|.
30. Tsou HC, Ping XL, Xie XX, et al. The genetic basis of Cowden's syndrome: three novel mutations in PTEN/MMAC1/TEP1. Hum Genet 1998;**102**(4):467-73
31. Orrico A, Galli L, Buoni S, et al. Novel PTEN mutations in neurodevelopmental disorders and macrocephaly. Clinical genetics 2009;**75**(2):195-8 doi: 10.1111/j.1399-0004.2008.01074.x[published Online First: Epub Date]|.
32. De Vivo I, Gertig DM, Nagase S, et al. Novel germline mutations in the PTEN tumour suppressor gene found in women with multiple cancers. J Med Genet 2000;**37**(5):336-41
33. McBride KL, Varga EA, Pastore MT, et al. Confirmation study of PTEN mutations among individuals with autism or developmental delays/mental retardation and macrocephaly.

Autism research : official journal of the International Society for Autism Research 2010;**3**(3):137-41 doi: 10.1002/aur.132[published Online First: Epub Date]|.
34. Kersseboom R, Dubbink HJ, Corver WE, et al. PTEN in colorectal cancer: a report on two Cowden syndrome patients. Clinical genetics 2012;**81**(6):555-62 doi: 10.1111/j.1399-0004.2011.01639.x[published Online First: Epub Date]|.
35. Nelen MR, van Staveren WC, Peeters EA, et al. Germline mutations in the PTEN/MMAC1 gene in patients with Cowden disease. Human molecular genetics 1997;**6**(8):1383-7
36. Elia M, Amato C, Bottitta M, et al. An atypical patient with Cowden syndrome and PTEN gene mutation presenting with cortical malformation and focal epilepsy. Brain Dev 2012;**34**(10):873-6 doi: 10.1016/j.braindev.2012.03.005[published Online First: Epub Date]|.
37. Liaw D, Marsh DJ, Li J, et al. Germline mutations of the PTEN gene in Cowden disease, an inherited breast and thyroid cancer syndrome. Nature Genetics 1997;**16**(1):64-67
38. Kurose K, Araki T, Matsunaka T, et al. Variant manifestation of Cowden disease in Japan: hamartomatous polyposis of the digestive tract with mutation of the PTEN gene. Am J Hum Genet 1999;**64**(1):308-10 doi: 10.1086/302207[published Online First: Epub Date]|.
39. Heindl M, Handel N, Ngeow J, et al. Autoimmunity, intestinal lymphoid hyperplasia, and defects in mucosal B-cell homeostasis in patients with PTEN hamartoma tumor syndrome. Gastroenterology 2012;**142**(5):1093-96 e6 doi: 10.1053/j.gastro.2012.01.011[published Online First: Epub Date]|.
40. O'Roak BJ, Vives L, Fu W, et al. Multiplex targeted sequencing identifies recurrently mutated genes in autism spectrum disorders. Science 2012;**338**(6114):1619-22 doi: 10.1126/science.1227764[published Online First: Epub Date]|.
41. Derrey S, Proust F, Debono B, et al. Association between Cowden syndrome and Lhermitte-Duclos disease: report of two cases and review of the literature. Surg Neurol 2004;**61**(5):447-54; discussion 54 doi: 10.1016/S0090-3019(03)00576-7[published Online First: Epub Date]|.
42. Tekin M, Hismi BO, Fitoz S, et al. A germline PTEN mutation with manifestations of prenatal onset and verrucous epidermal nevus. American journal of medical genetics Part A 2006;**140**(13):1472-5 doi: 10.1002/ajmg.a.31273[published Online First: Epub Date]|.
43. Busa T, Chabrol B, Perret O, et al. Novel PTEN germline mutation in a family with mild phenotype: difficulties in genetic counseling. Gene 2013;**512**(2):194-7 doi: 10.1016/j.gene.2012.09.134[published Online First: Epub Date]|.
44. Boccone L, Dessi V, Zappu A, et al. Bannayan-Riley-Ruvalcaba syndrome with reactive nodular lymphoid hyperplasia and autism and a PTEN mutation. American journal of medical genetics Part A 2006;**140**(18):1965-9 doi: 10.1002/ajmg.a.31396[published Online First: Epub Date]|.
45. Kubo Y, Urano Y, Hida Y, et al. A novel PTEN mutation in a Japanese patient with Cowden disease. Br J Dermatol 2000;**142**(6):1100-5
46. Gicquel JJ, Vabres P, Bonneau D, et al. Retinal angioma in a patient with Cowden disease. Am J Ophthalmol 2003;**135**(3):400-2
47. Nelen MR, Kremer H, Konings IB, et al. Novel PTEN mutations in patients with Cowden disease: absence of clear genotype-phenotype correlations. European journal of human genetics : EJHG 1999;**7**(3):267-73 doi: 10.1038/sj.ejhg.5200289[published Online First: Epub Date]|.
48. Banneau G, Guedj M, MacGrogan G, et al. Molecular apocrine differentiation is a common feature of breast cancer in patients with germline PTEN mutations. Breast cancer research : BCR 2010;**12**(4):R63 doi: 10.1186/bcr2626[published Online First: Epub Date]|.
49. Marsh DJ, Dahia PL, Zheng Z, et al. Germline mutations in PTEN are present in Bannayan-Zonana syndrome [letter]. Nat Genet 1997;**16**(4):333-4
50. Lachlan KL, Lucassen AM, Bunyan D, et al. Cowden syndrome and Bannayan Riley Ruvalcaba syndrome represent one condition with variable expression and age-related penetrance: results of a clinical study of PTEN mutation carriers. J Med Genet 2007;**44**(9):579-85 doi: 10.1136/jmg.2007.049981[published Online First: Epub Date]|.

51. Kirches E, Steiner J, Schneider T, et al. Lhermitte-Duclos disease caused by a novel germline PTEN mutation R173P in a patient presenting with psychosis. Neuropathol Appl Neurobiol 2010;**36**(1):86-9 doi: 10.1111/j.1365-2990.2009.01041.x[published Online First: Epub Date]|.
52. Thiffault I, Schwartz CE, Der Kaloustian V, et al. Mutation analysis of the tumor suppressor PTEN and the glypican 3 (GPC3) gene in patients diagnosed with Proteus syndrome. American journal of medical genetics Part A 2004;**130A**(2):123-7 doi: 10.1002/ajmg.a.30335[published Online First: Epub Date]|.
53. Kim DK, Myung SJ, Yang SK, et al. Analysis of PTEN gene mutations in Korean patients with Cowden syndrome and polyposis syndrome. Dis Colon Rectum 2005;**48**(9):1714-22 doi: 10.1007/s10350-005-0130-9[published Online First: Epub Date]|.
54. Staal FJ, van der Luijt RB, Baert MR, et al. A novel germline mutation of PTEN associated with brain tumours of multiple lineages. Br J Cancer 2002;**86**(10):1586-91 doi: 10.1038/sj.bjc.6600206[published Online First: Epub Date]|.
55. Chi SG, Kim HJ, Park BJ, et al. Mutational abrogation of the PTEN/MMAC1 gene in gastrointestinal polyps in patients with Cowden disease. Gastroenterology 1998;**115**(5):1084-9
56. Sawada T, Hamano N, Satoh H, et al. Mutation analysis of the PTEN / MMAC1 gene in Japanese patients with Cowden disease. Jpn J Cancer Res 2000;**91**(7):700-5
57. Lynch ED, Ostermeyer EA, Lee MK, et al. Inherited mutations in PTEN that are associated with breast cancer, cowden disease, and juvenile polyposis. Am J Hum Genet 1997;**61**(6):1254-60 doi: 10.1086/301639[published Online First: Epub Date]|.
58. Davidson L, Maccario H, Perera NM, et al. Suppression of cellular proliferation and invasion by the concerted lipid and protein phosphatase activities of PTEN. Oncogene 2010;**29**(5):687-97 doi: onc2009384 [pii] 10.1038/onc.2009.384[published Online First: Epub Date]|.
59. McConnachie G, Pass I, Walker SM, et al. Interfacial kinetic analysis of the tumour suppressor phosphatase, PTEN: evidence for activation by anionic phospholipids. Biochem J 2003;**371**(Pt 3):947-55
